# How the Chemical Properties of Polysaccharides Make It Possible to Design Various Types of Organic–Inorganic Composites for Catalytic Applications

**DOI:** 10.3390/molecules29133214

**Published:** 2024-07-06

**Authors:** Sandugash Akhmetova, Alima Zharmagambetova, Eldar Talgatov, Assemgul Auyezkhanova, Makpal Malgazhdarova, Murat Zhurinov, Arlan Abilmagzhanov, Aigul Jumekeyeva, Alima Kenzheyeva

**Affiliations:** Laboratory of Organic Catalysis, D.V. Sokolsky Institute of Fuel, Catalysis, and Electrochemistry, Kunaev Str. 142, Almaty 050010, Kazakhstan; s.akhmetova@ifce.kz (S.A.); zhalima@mail.ru (A.Z.); e.talgatov@ifce.kz (E.T.); m.malgazhdarova@ifce.kz (M.M.); m.zhurinov@ifce.kz (M.Z.); a.abilmagzhanov@ifce.kz (A.A.); jumekeeva@mail.ru (A.J.); a.kenzheeva@ifce.kz (A.K.)

**Keywords:** polysaccharide, chitosan, starch, pectin, cellulose, catalyst

## Abstract

Recently, the use of plant-origin materials has become especially important due to the aggravation of environmental problems and the shortage and high cost of synthetic materials. One of the potential candidates among natural organic compounds is polysaccharides, characterized by a number of advantages over synthetic polymers. In recent years, natural polysaccharides have been used to design composite catalysts for various organic syntheses. This review is devoted to the current state of application of polysaccharides (chitosan, starch, pectin, cellulose, and hydroxyethylcellulose) and composites based on their catalysis. The article is divided into four main sections based on the type of polysaccharide: (1) chitosan-based nanocomposites; (2) pectin-based nanocomposites; (3) cellulose (hydroxyethylcellulose)-based nanocomposites; and (4) starch-based nanocomposites. Each section describes and summarizes recent studies on the preparation and application of polysaccharide-containing composites in various chemical transformations. It is shown that by modifying polysaccharides, polymers with special properties can be obtained, thus expanding the range of biocomposites for catalytic applications.

## 1. Introduction

The current requirements for cleaner technologies represent the most important challenge on the journey toward more sustainable and environmentally friendly chemistry. In order to achieve progress in the development of clean technologies, attempts are being made to avoid the use of toxic and hazardous substances and replace fossil resources with alternative natural raw materials. Polysaccharides, as one of the most widespread natural organic compounds possessing several advantages over synthetic polymers, attract much attention. They are safe, economical, stable, biocompatible, and biodegradable, and they easily interact with metals due to their high functionality [1,2,3]. Numerous hydroxyl (–OH), carboxyl (–COOH), or amine (–NH_2_) groups covalently anchored to the periphery of the polysaccharide glucose units provide them with a versatile ability for modification and functionalization to obtain materials with useful properties. Recently, they have been successfully used to design novel green materials for application in different fields such as pharmacology, medicine, the food sector, cosmetics, the chemical industry, catalysis, and remediation [3,4,5,6,7,8,9,10,11,12,13,14]. Each of the existing areas is currently being developed intensively and therefore requires separate detailed analyses and summaries regarding the results.

Catalysis plays a key role in the development of green technologies for chemicals and materials. One of the potential applications of polysaccharides is the development of biocomposite catalysts for various organic syntheses.

In recent decades, the number of publications (including reviews [13,14,15]) on the design and application of such catalysts has increased significantly [14,15,16,17,18,19,20,21,22,23,24,25,26,27,28,29,30,31,32,33,34]. The presence of the abovementioned functional groups in polysaccharides is a unique opportunity for the physicochemical binding of metal ions to form metal nanoparticles. The strong affinity of these biopolymers to interact with metal nanoparticles leads to stabilization and prevents metal leaching, which is an important factor in catalysis. Today, many chemical processes are tested using different types of metal–polysaccharide catalysts [14,15,16]. For example, chitosan-supported palladium catalysts have shown high activity, good reusability, and long life in the selective hydrogenation of different aromatic compounds, including aldehydes [17,18,19,32], nitroarenes into corresponding amines [20,21], and acetylenic compounds [22,23].

A comprehensive analysis of oxidation syntheses of nanocatalysts containing different types of polysaccharides is presented in [24]. Chitosan-based metal catalysts have been used successfully for the oxidation of organic compounds [24,25,26,27], the “click” cycloaddition of azides with terminal alkynes [28,29], and the cyclopropanation of olefins [29].

Cross-coupling reactions have gathered much attention over the past few decades as a simple and mild way to synthesize organic compounds. Palladium is one of the most effective catalysts for these syntheses. Various renewable polysaccharides have been used to support palladium catalysts for cross-coupling reactions [30,31,32,33,34,35,36]. The most commonly used is chitosan. The other polysaccharides used as components of catalytic systems are starch, pectin, cellulose and its derivatives, alginate, gum, etc. [36].

One of the main challenges in the field of catalysis is the preparation of new materials to replace traditional catalysts quickly, cheaply, and efficiently. From this point of view, polysaccharide-based catalysts are very promising. According to recent reviews [13,15,24,36], many approaches are used for the preparation of catalysts based on polysaccharides because various heterogeneous catalysts are prepared with a variety of polysaccharides using different techniques, and their performances are tested in different reactions. However, to our knowledge, there are no reviews dedicated to the classification of catalysts from the point of view of the chemical properties of each polysaccharide to allow for the design of various types of heterogeneous catalysts based on them.

It is difficult to discuss all the varieties of natural polysaccharides used in the development of novel catalytic systems and to analyze and classify the methods of their preparation and application in one review. We have focused on the analysis of the use of chitosan, cellulose, and their derivatives as the most abundant naturally occurring and commonly considered biopolymers in the design of catalysts. There is increasing interest in the study of starch and pectin as catalyst components due to the widespread methods for their extraction from plant waste and the possibility of forming physicochemical bonds with metal ions and inorganic supports.

In this review, we present some results on the application of these polysaccharides as components of catalysts for different organic processes, classify the composite catalysts into several types based on the chemical properties of each polysaccharide, and partly discuss the advantages and disadvantages of methods for their preparation and future trends.

## 2. Chitosan-Based Nanocomposites

**Figure 1:**
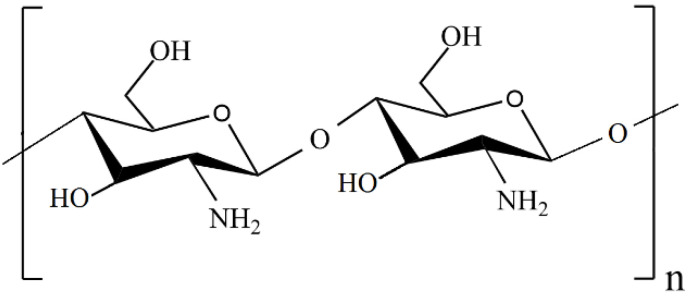
Structure of a chitosan molecule.

Recently, chitosan-based composites have attracted much attention due to the unique properties of chitosan, such as nontoxicity, renewability, and high availability [37,38]. Chitosan (CS or Chit) is a linear random copolymer of *N*-acetyl-*D*-glucosamine and deacetylated *D*-glucosamine units connected by *β*-1,4 glycosidic linkages (Figure 1), obtained from the chemical alkaline deacetylation of chitin [39,40].

Chitin is the second-most prevalent natural polysaccharide after cellulose [41,42], meaning that chitosan (CS) is also a very abundant biopolymer. Chitosan has three types of reactive functional groups, i.e., an amino/acetamido group and both primary and secondary hydroxyl groups at the C-2, C-3, and C-6 positions, respectively [39,43]. The degree of deacetylation is one of the most important parameters affecting the properties of chitosan, such as its solubility in aqueous acidic solutions, the degree of swelling in water, crystallinity, average molecular weight, susceptibility to biodegradation, biological activity, and biocompatibility [41]. The free amine groups (–NH_2_), located at C-2 on the rings of the *D*-glucosamine repeated units, may be pronated under acidic conditions [39]. At pH < 6.3, the free amino groups are protonated and positively charged, and chitosan is a water-soluble cationic polyelectrolyte. At pH > 6.3, the free amino groups become deprotonated, and the chitosan loses its charge and becomes insoluble [44]. Due to the presence of free amino and hydroxyl groups, chitosan is more reactive, which means that it is susceptible to chemical modifications. For example, chitosan forms chelate complexes with metal ions. The free amino groups in chitosan are considered to be much more effective for metal ion complexation than the acetyl groups in chitin [41]. Thus, chitosan exhibits cationic behavior in acidic solutions and a strong affinity for metal ions; therefore, it has received increased attention for the preparation of heterogeneous catalysts as both chelating and stabilizing agents [45,46].

### 2.1. Chitosan as a Catalyst Support or Stabilizing Agent

Due to the presence of reactive amino groups, chitosan’s thermal stability and insolubility in organic solvents hold great potential, representing a versatile platform for supporting metal ions (or nanoparticles) [46]. Transition metal ions, such as palladium, ruthenium, gold, silver, copper, cobalt, and iron, immobilized on chitosan have found applications as catalysts for hydrogenation, coupling, oxidation, and other organic synthesis reactions [29,46]. There are various methods for the preparation of transition metal catalysts supported on chitosan.

**Figure 2:**
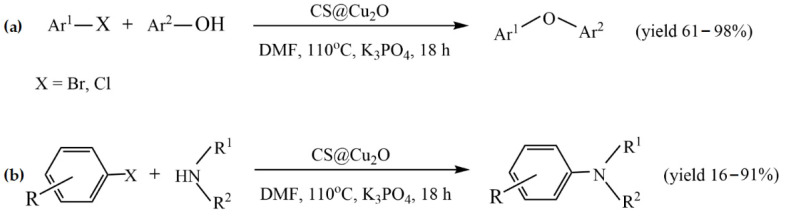
CS@Cu_2_O-catalyzed coupling reactions of (**a**) aryl bromide/chloride with phenols and (**b**) of *N*-heterocyclic amines with aryl bromide/iodides. Reprinted with permission from ref. [47]. Copyright 2019 Science China Press.

The simplest method to prepare chitosan-based metal catalysts is to mix a slurry of chitosan with a solution of the metal precursor, resulting in the adsorption of metal ions. Cunwei et al. [47] used this approach to prepare a series of chitosan-supported Cu catalysts for ligand-free C-O and C-N coupling reactions. The catalysts differ according to the metal precursor and solvent used for their preparation. Chitosan (CS)-supported copper salts (CS@CuSO_4_ and CS@Cu(OAc)_2_) were prepared by suspending CS in an aqueous solution of CuSO_4_ and Cu(OAc)_2_. A CS@CuI catalyst was prepared via the adsorption of CuSO_4_ on chitosan in methanol media, followed by treating the resulting blue solid with NaI. The ultrasonic stirring of Cu_2_O nanoparticles and chitosan in toluene resulted in CS@Cu_2_O, which showed better catalytic potency in the coupling of bromobenzene with phenol than other Cu catalysts. The best yield (95%) for this catalyst was achieved at the following reaction conditions: 2.0 mmol of phenol, 1.0 mmol of ArBr, 0.5 mol% CS@Cu_2_O, 3.0 mmol of K_3_PO_4_, 0.5 mL of anhydrous DMF under an Ar atmosphere for 18 h. This catalyst was also studied in other C-O and C-N coupling reactions, providing good and excellent yields under the same reaction conditions (Figure 2). In addition, the catalyst was reused in the coupling reaction of phenol with chlorobenzene during four runs without a significant decrease in its catalytic activity, and no leaching of copper was observed [47].

Another simple method to prepare chitosan-based metal catalysts is the physical mixing of chitosan and metal salts. In [20], Pd/Chit catalysts were prepared via the physical mixing of chitosan (Chit), PdCl_2_, NaOH, and ascorbic acid (AA) using a mortar and pestle. The AA served as a mild reducing agent in the conversion of PdCl_2_ to Pd nanoparticles (NPs), and the NaOH was crucial in anchoring the resulting Pd NPs to the chitosan support during solid grinding. The resulting catalyst demonstrated a higher available surface area and a larger number of accessible active sites than other Pd NP catalysts reported using solution methods. As a result, in the presence of NaBH_4_ (10 mmol) as a hydrogen source, Pd/Chit (150 mg) demonstrated enhanced catalytic activity (TOF = 8498 h^−1^; yield = 96%) for the reduction of *p*-nitrophenol (1 mmol) to p-aminophenol in water (10 mL) at 300 K. The catalyst was reused four to ten times without a significant decrease in its catalytic activity. Furthermore, a kinetics investigation of the reduction of nitroarene under optimal reaction conditions showed that Pd/Chit has a substantially lower activation energy than other reported Pd NP catalysts.

**Figure 3:**
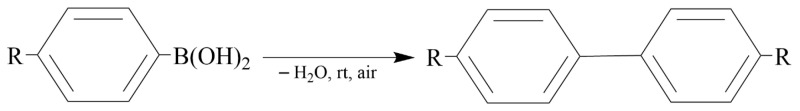
The homocoupling of arylboronic acid reaction.

Reddy et al. [48] used a solid-grinding approach for the synthesis of gram-scale quantities of solid chitosan-supported gold NPs using sodium borohydride (NaBH_4_) as the reducing agent (Figure 3). It was found that gold NP sizes could be modulated (2.8–9.5 nm) by varying the amount of gold NPs (0.47–12.5 wt.%) loaded onto the solid chitosan support. These solid chitosan-supported gold NPs were used as heterogeneous catalysts for the oxidative homocoupling of arylboronic acids (0.25 mmol) into biphenyls and the aerobic oxidation of benzyl alcohols (0.25 mmol) into the corresponding aldehydes and acids in water (15 mL) at room temperature and under open air. The catalysts showed higher activity and selectivity for the homocoupling of arylboronic acid reactions, yielding 94–99% biphenyls, when compared with those of other well-known hydrophilic polymer-stabilized colloidal nanogold catalysts prepared using conventional solution-based methods.

**Figure 4:**
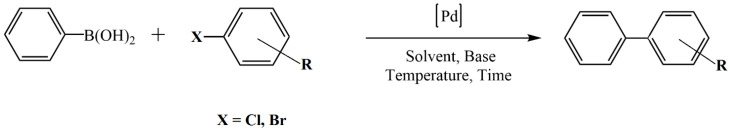
Suzuki–Miyaura cross-coupling of phenylboronic acid with different aryl iodides/bromides.

However, the preparation of chitosan-based metal catalysts from a biopolymer dilute acidic solution is still the preferred and more common approach. Chitosan is capable of adsorbing and stabilizing large amounts of metal ions. Furthermore, chitosan has an intrinsic ability to reduce metal ions to metal particles. Catalyst materials may also be fabricated by mixing chitosan with preformed metal particles [29]. Grandini et al. [49] prepared palladium nanoparticles supported on a chitosan/cellulose film by stirring microgranular cellulose and previously prepared Pd NPs in a viscous acetic acid aqueous solution of chitosan, followed by drying the obtained homogenous mixture in a glass Petri plate. The resulting fibrous catalyst employed an adequate palladium load (0.5 mol%) and provided a very good to high product yield (78–98%) in the Suzuki–Miyaura cross-coupling of phenylboronic acid (1.5 mmol) with different aryl iodides/bromides (1 mmol) in presence of K_2_CO_3_ (2 mmol) as a base in ethanol (3 mL) at 100 °C (Figure 4). In addition, the catalyst demonstrated excellent recyclability in the cross-coupling of phenylboronic acid with 4-iodoanisole. The catalyst was reused during 10 runs without a decrease in catalytic activity.

The chemical properties of chitosan allow for the fabrication of catalysts in various forms, such as gels, spheres, fibers, etc. The preparation of such catalysts is accompanied by the precipitation of chitosan with an alkali or its interaction with cross-linking agents. It should be noted that the precipitation of chitosan in the presence of transition metal salts or nonmetal/metal oxides (SiO_2_, Al_2_O_3_, Fe_3_O_4_, etc.) results in the formation of metal (or nonmetal) oxides stabilized/functionalized with the polymer, where an inorganic component is responsible for any catalytic functions or serves as the core hybrid support material for the separation of a catalyst (transition metal ions or NPs, enzymes, etc.) immobilized on the polymer shell (see Section 2.2 and Section 2.3). Schiff’s base reaction between the amino groups of chitosan and aldehydes improves the mechanical properties of catalytic microspheres due to the cross-linking of the polymer and results in the formation of new functional groups with a better affinity for metal ions; this can decrease the leaching of metal ions from the cross-linked polymer matrix and increase the lifetime of a catalyst. For this reason, chitosan can also be modified with other organic molecules (see Section 2.3).

**Figure 5:**
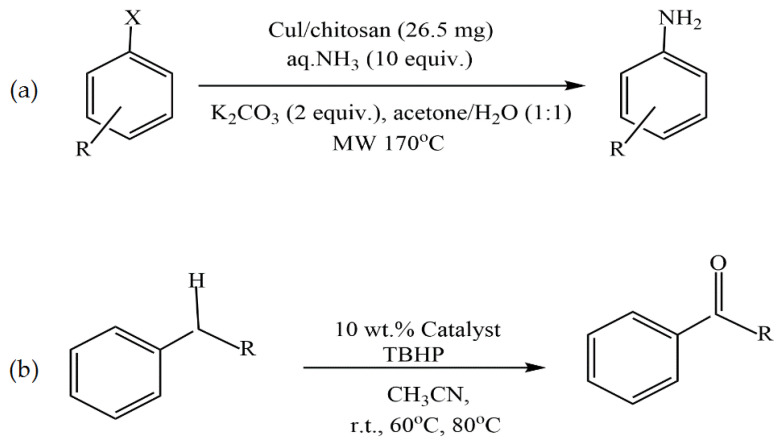
C-X amination of aryl halides with ammonia (**a**) and C-H oxidation of alkylarenes to ketones with tert-butyl hydroperoxide (TBHP) (**b**).

Kanarat et al. [50] reported that the dropwise addition of a solution containing chitosan and copper salt (Cu(OAc)_2_ or CuI) into a NaOH solution resulted in spherical granules of Cu/chitosan catalysts for the C-H oxidation of alkylarenes to ketones with tert-butyl hydroperoxide (TBHP) and the C-X amination of aryl halides with ammonia (Figure 5). These catalysts were compatible with various substrates, resulting in 2–97% and 39–99% yields for the oxidation and amination reactions, respectively. Moreover, chitosan served as both a versatile support to enhance the catalytic performance of copper-active species and a base to promote the catalytic reactions.

Correia et al. prepared calcium/chitosan spheres via the dropwise addition of a chitosan and calcium nitrate solution into an aqueous ammonia solution [51]. Then, these calcium-incorporated chitosan spheres were calcined in order to obtain a porous calcium catalyst without organic material. Afterward, the calcined calcium/chitosan spheres were used in the transesterification reaction of sunflower oil with methanol. The conversion of sunflower oil to methyl esters under optimized reaction conditions (oil/methanol ratio—1:9; catalyst amount—3 wt.%; time—4 h; temperature—60 °C; magnetic stirring—1000 rpm), which were determined by a factorial experimental design, was 56.12 ± 0.32 wt.%. These results show that chitosan can be used as a structuring agent in the fabrication of porous alkaline catalysts for biodiesel production.

**Figure 6:**
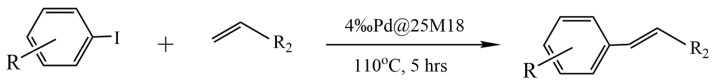
Pd@25M18-catalyzed Mizoroki–Heck reaction of aromatic iodides with olefins.

In [52], chitosan microsphere-supported palladium catalysts (Pd@CM) for a Mizoroki–Heck reaction were successfully prepared by electrospraying a PdCl_2_/chitosan mixture in a trifluoracetic acid (TFA) aqueous solution into a NaOH aqueous solution, followed by cross-linking the obtained polymer microspheres with glutaraldehyde. This novel heterogeneous palladium catalyst has been demonstrated to be an efficient catalyst for the Mizoroki–Heck reaction of aromatic iodides with olefins (Figure 6). The reaction yield in the presence of triethylamine in *N*,*N*-dimethylacetamide at 110 °C reached 82–98%. The high catalytic activity of Pd@CM has been attributed to the high dispersion of palladium species and the small size of chitosan microspheres, and the remarkable stability and recyclability can be ascribed to the chelation of palladium with the abundant amine and hydroxyl groups on the chitosan chain and the entrapment of palladium species in the cross-linked chitosan chain. It should be noted that Pd@CM was even more active than the catalyst with palladium species adsorbed on the surface of chitosan microspheres (Pd-CM).

**Figure 7:**
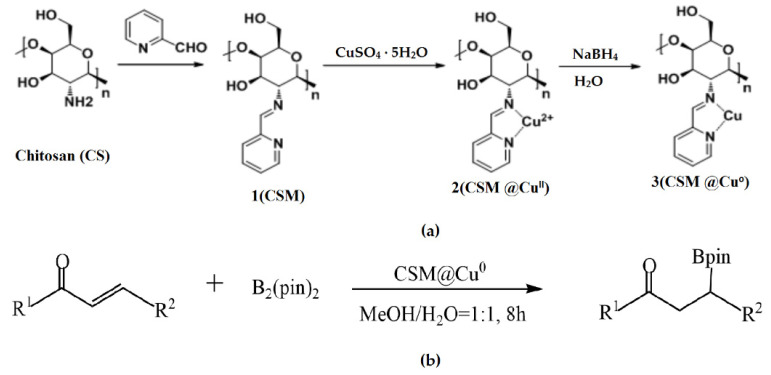
Preparation of the CSM@Cu^0^ catalyst (**a**) and its application to the boron addition reactions (**b**). Reprinted from ref. [53].

Wang et al. used modified chitosan microspheres (CSMs) prepared by cross-linking a polymer with 2-pyridinecarboxaldehyde in a water/oil emulsion in the construction of a heterogeneous catalyst loaded with nano-Cu prepared using a reduction reaction (Figure 7a) [53]. Under mild conditions, such as no ligand at room temperature, the CSM@Cu^0^ catalyst was successfully applied to catalyze the borylation of α,β-unsaturated receptors in a water–methanol medium, yielding 17–100% of the corresponding β-hydroxy product (Figure 7b). Even after being repeatedly used five times, the catalyst still exhibited excellent catalytic activity.

**Figure 8:**
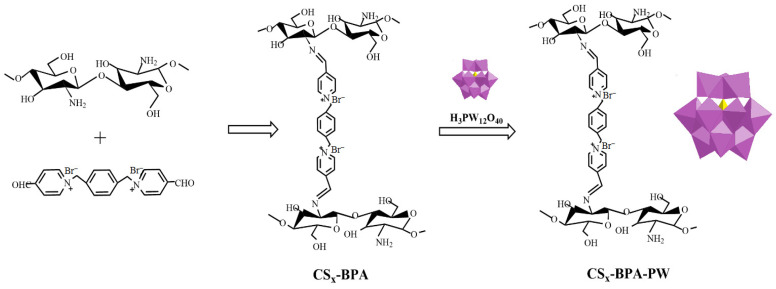
Schematic illustration outlining the preparation and structure of the CS_x_-BPA-PW catalysts [54].

Li et al. [54] demonstrated that cross-linked chitosan (CS) bearing –NH_2_ functional groups obtained by cross-linking CS with a dicationic pyridine ionic compound (BPA) acted as a promising support to produce heterogeneous H_3_PW_12_O_40_ catalysts (Figure 8). The redox properties of polyoxometalate (PW) anions could be controlled by varying the amount of CS in CS_x_-BPA-PW. The resulting hybrid, CS_1.5_-BPA-PW (0.01 mmol), performed as an efficient solid catalyst in the epoxidation of cyclooctene (5 mmol substrate; 10 mmol H_2_O_2_; 5 mL acetonitrile; 70 °C; 1 h; conversion: 100%; selectivity for the cyclooctene oxide: 100%) and the oxidation of benzyl alcohol (5 mmol substrate; 6 mmol H_2_O_2_; 5 mL acetonitrile; 90 °C; 4 h; conversion: 88%; selectivity for the benzaldehyde: 97%) with H_2_O_2_.

### 2.2. Chitosan-Modified Catalysts on Inorganic Supports

The immobilization and stabilization of metal nanoparticles on chitosan (CS or Chit) lead to the formation of two-component composites with tunable catalytic properties due to the effect of the polymer on the size, shape, morphology, and other physicochemical characteristics of active species. At the same time, the use of chitosan as a support in catalysis is troubled by problems such as insufficient resistance to attrition and the small specific surface area of the polymer. One of the approaches for overcoming these problems is using inorganic materials (SiO_2_, metal oxides, and clays) modified with chitosan as a catalytic support. Such composite supports can combine the advantages of both chitosan (the chelation and stabilization of metal species) and inorganic material (high surface area and mechanical and thermal stability). There are various methods to obtain three-component metal–chitosan–inorganic material catalysts.

**Figure 9:**
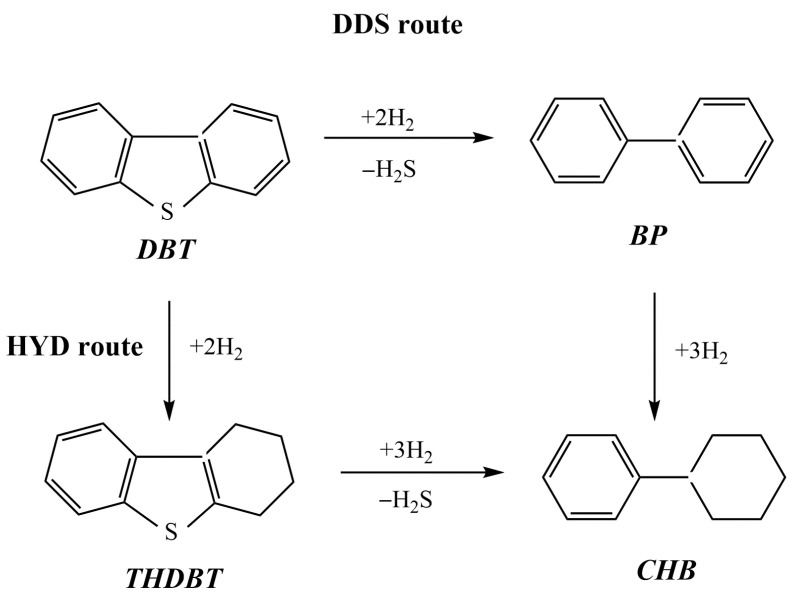
Reaction network of the dibenzothiophene (DBT) hydrodesulfurization process.

In [55], two series of NiMoP catalysts supported on chitosan-modified alumina (A) and alumina titania (AT) were prepared by using a sequential pore-filling impregnation procedure. In each series, the order of chitosan and metal precursor impregnation was varied in the same way. The catalysts were studied in the hydrodesulfurization (HDS) of dibenzothiophene (DBT, 0.3 g) in a batch reactor using n-hexadecane (100 mL) as the solvent at 320 °C, 72.4 kg cm^−2^, and 1000 rpm. The HDS reaction can occur through both the direct desulfurization (DDS) and hydrogenation (HYD) pathways, with the formation of the following products: biphenyl (BP), cyclohexylbenzene (CHB), tetrahydrodibenzothiophene (THDBT), and bicyclohexane (BCH) (Figure 9). The series of catalysts supported on alumina presented the most remarkable effect of chitosan, in which the –OH and –NH groups of the organic molecule interacted with the acid sites of the support, weakening the interaction between alumina and the deposited metal phases. In all cases, DBT was converted mainly through direct sulfur removal. The catalysts ChP3/A (alumina support impregnated with chitosan in phosphoric acid solution prior to NiMoP deposition) and ChP4/AT (alumina titania support impregnated with NiMoP solution prior to contact with a solution comprising chitosan and phosphorus) exhibited the best performance in HDS reactions and also showed the highest selectivity in biphenyl formation (81.4% and 88.6%, respectively). The presence of carbonaceous residuals on the catalyst’s surface, as shown via XPS, could enhance the HDS activity from the ChP4/AT sample.

**Figure 10:**
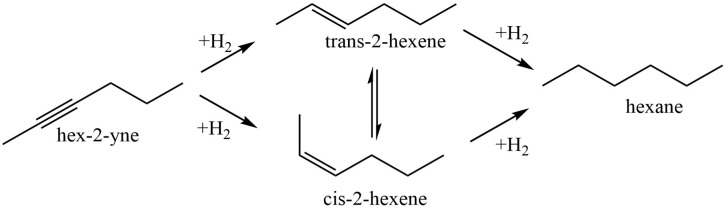
Reaction network of n-hex-2-yne hydrogenation.

Sun et al. [56] used chitosan (CS) and polyvinyl pyrrolidinone (PVP) in order to stabilize Pd nanoparticles on the surface of modified montmorillonite clay (AlMn-PM). The preparation of Pd@CS/AlMn-PM and Pd@PVP/AlMn-PM catalysts was accomplished via the sequential adsorption of a polymer (CS or PVP) and palladium ions on pillared montmorillonite clay with further heat treatment of the resulting three-component composites at 200 °C. The resultant Pd@polymer/AlMn-PM heterogeneous catalytic nanocomposites (2.3 µmol of Pd) showed highly competitive catalytic performances in Sonogashira coupling reactions with various aryl iodides (1 mmol) and phenylacetylene (1.2 mmol) at 90 °C for 1 h (yield 92–100%). Pd@CS/AlMn-PM showed better comprehensive catalytic performance than Pd@PVP/AlMn-PM. This was mainly attributed to its higher specific area, stronger chelation of Pd species, and better solvent resistance. In [57], Pd and PdAg catalysts supported on chitosan (Chit)-modified zinc oxide were prepared using the same method (sequential adsorption), except that the resulting Pd-Chit/ZnO and PdAg-Chit/ZnO composites were not heat treated and were studied in n-hex-2-yne hydrogenation at 40 °C and P = 0.1 MPa (Figure 10). A comparison of the catalysts in the hydrogenation process indicated that the catalytic properties of such composites could be tuned by varying the chitosan content and adding a second metal. A monometallic 1% Pd-Chit/ZnO catalyst with 2 wt.% chitosan content ([Pd]:[Chit] = 1:1.3 mol/mol) showed the highest activity in the n-hex-2-yne hydrogenation, with a hydrogen uptake rate of 21 × 10^−6^ mol/s. A further increase in the polymer content ([Pd]:[Chit] = 1:3.3 mol/mol) or the addition of silver with a reduction in the palladium content promoted an increase in the yield of the target product (selectivity to cis-2-hexene increased from 82% to 91–96%), and, at the same time, the activity of the catalysts was reduced.

**Figure 11:**
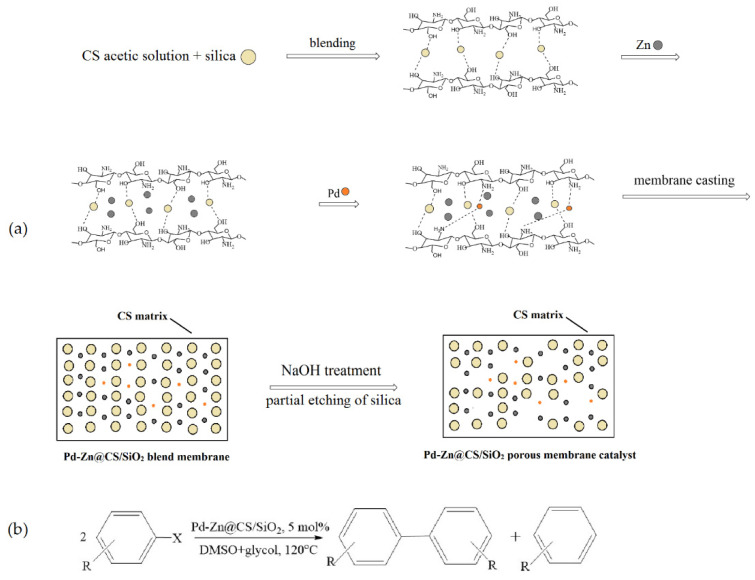
Preparation of the Pd-Zn@CS/SiO_2_ membrane catalyst (**a**) and its application for the reductive homocoupling of various aryl halides (**b**). Adapted from [58].

Liu et al. [58] synthesized an efficient heterogeneous catalyst, which included palladium and zinc nanoparticles supported on a chitosan/silica (CS/SiO_2_) composite membrane. The catalyst was prepared by casting a mixture containing chitosan in an acetic acid aqueous solution, silica colloids (40 wt.%), Zn powder, and a Na_2_PdCl_4_ solution, followed by partially etching SiO_2_ from the resulting membrane (Figure 11a). The high-resolution transmission electron microscopy and X-ray photoelectron spectroscopy characterization of this catalyst showed that the Pd^0^ nanoparticles (below 5 nm) and Zn^0^ aggregates (about 10–15 nm) were dispersed well in the CS/SiO_2_ matrix. The prepared Pd-Zn@CS/SiO_2_ membrane catalyst was highly active for the Ullmann reductive homocoupling reactions of various aryl halides (41.8–100% conversion), and the selectivity for biphenyls reached 54–98% (Figure 11b). The catalyst could be recycled five times without significant loss of activity.

In [59], monometallic Cu and bimetallic Cu-M (M = Co, Zn, Fe, Ni) catalysts were prepared using the NaOH-assisted precipitation of chitosan (CS) on silica (SiO_2_) and the further immobilization of metal ions on the resulting CS-SiO_2_ composite. The Cu@CS-SiO_2_ and Cu-M@CS-SiO_2_ catalysts demonstrated high efficiency in the process of catalytic wet peroxide oxidation for the degradation of 1,1-dimethyl hydrazine wastewater with H_2_O_2_ (100% in 10 min) at 65 °C. The Cu-Zn@CS-SiO_2_ catalyst activity was minimally affected within six reaction cycles.

**Figure 12:**
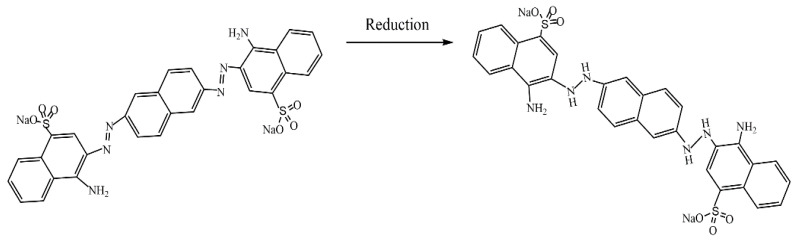
Reductive decolorization of Congo red using NaBH_4_ as a reductive agent in ambient conditions.

Ali et al. [60] also used the ability of chitosan to precipitate at pH > 6.3 in order to obtain a chitosan–silica (CH-SiO_2_) nanocomposite fiber as a support material for metal nanoparticles (Cu, Co, Ag, and Ni). The CH-SiO_2_ nanocomposite fiber was prepared by adding a mixture of SiO_2_ nanoparticles dispersed in a chitosan solution to a concentrated aqueous solution of NaOH using a clean disposable syringe. Metal nanoparticles (NPs) templated on chitosan–silica nanocomposite fiber were prepared via the treatment of CH-SiO_2_ with a metal salt solution and NaBH_4_. The catalysts were studied in both the reduction of 4-nitroaniline (4-NA) and the decolorization of Congo red (CR) (Figure 12) using NaBH_4_ as a reductive agent under ambient conditions. The Cu nanoparticle (NP)-loaded CH-SiO_2_ (Cu/CH-SiO_2_) showed high catalytic efficiency in the reduction of 4-NA and CR when compared to other loaded metal NPs. Apparent rate constants of 6.17 × 10^−3^ s^−1^ and 1.68 × 10^−2^ s^−1^ and turnover frequencies (TOFs) of 4.693 h^−1^ and 3.965 h^−1^ were observed for the reduction of 4-NA and CR, respectively. In addition, the catalytic activity of the Cu/CH-SiO_2_ catalyst was also examined and found to be efficient in the reduction of nitrophenols (2-NP, 3-NP, and 4-NP) and other dyes.

**Figure 13:**
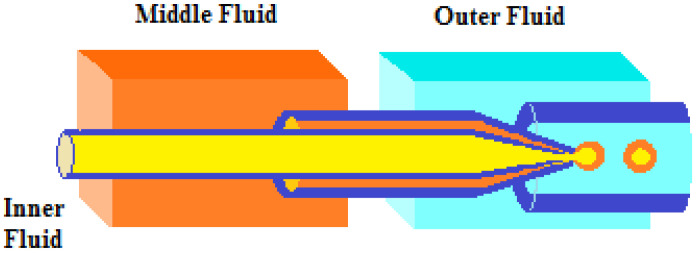
Schematic diagram of a micro fluidic device [61].

Ma et al. [61] proposed a simple microfluidic method to prepare monodispersed palladium nanoparticles (NPs) supported on silica/chitosan core–shell hybrid microspheres (Figure 13). In order to prepare the silica/chitosan core–shell hybrid microspheres, the inner (SiO_2_ sol) and middle (chitosan aqueous solution with acetic acid) fluids were injected (in the dispersed phase) into the microchannel and broken into monodispersed droplets under the shearing force of the continuous flow at the intersection. The droplets (a droplet of SiO_2_ sol inside a droplet of chitosan solution) were formed in a Teflon tube and collected in a solidification bath, where Schiff’s base reaction between chitosan and glutaraldehyde and the extraction of water from droplets in n-octanol were used to presolidify the droplets. In order to obtain the catalyst, palladium ions were adsorbed onto the shell layer of the microspheres via chelation and were then reduced to Pd(0) using NaBH_4_ at room temperature. Because a large amount of the –NH_2_ provided by chitosan has a strong force with metal ions, the Pd nanoparticles loaded on the microspheres remained monodispersed and had a mean diameter of only 2–3 nm. In addition, the core–shell structure indicated that chitosan had mainly been distributed on the shell of the microspheres, indicating that palladium was mainly loaded on the shell, too, which can effectively reduce the mass transfer distance between the reactant and catalyst. Therefore, the supported Pd catalyst showed superb catalytic performance in the hydrogenation of cyclohexene (TOF 5.89 min^−1^) under the optimal conditions of a H_2_/liquid ratio of 25, a Pd loading amount of 30 mg/g, 300 K, and P = 0.1 MPa. Furthermore, the silica/chitosan-supported catalyst showed excellent stability, with relatively high catalytic performance for 7 days without an obvious decrease in its activity.

### 2.3. Chitosan-Based Magnetic Catalysts

Magnetic iron oxide nanoparticles (MNPs) represent a promising material for catalytic applications due to their inherent unique properties, such as a high specific surface area, ordered mesopore structures, high loading of complex immobilization, relative chemical inertness, easy dispersibility in solutions, and magnetic properties [62,63]. Immobilizing a catalyst on the surface of MNPs allows for the simple separation of the magnetic catalyst without the need for centrifugation, filtration, or other tedious methods [64]. However, magnetic nanoparticles (MNPs) themselves have some disadvantages, such as being prone to self-oxidation and agglomeration, having intrinsic instability, and exhibiting magnetic dipole interactions, resulting in the significant deterioration of inherent unique nanoparticle properties [65]. Therefore, coating MNPs with the appropriate agents before using the immobilizing catalyst is required to overcome these disadvantages. Chitosan is one of the most promising coating agents that can provide a number of benefits in the design of magnetic catalysts. More specifically, the unique chemical properties of chitosan allow for the simple functionalization of MNPs, and diverse types of catalysts for various reactions can be obtained.

#### 2.3.1. Chitosan as a Catalyst

**Figure 14:**
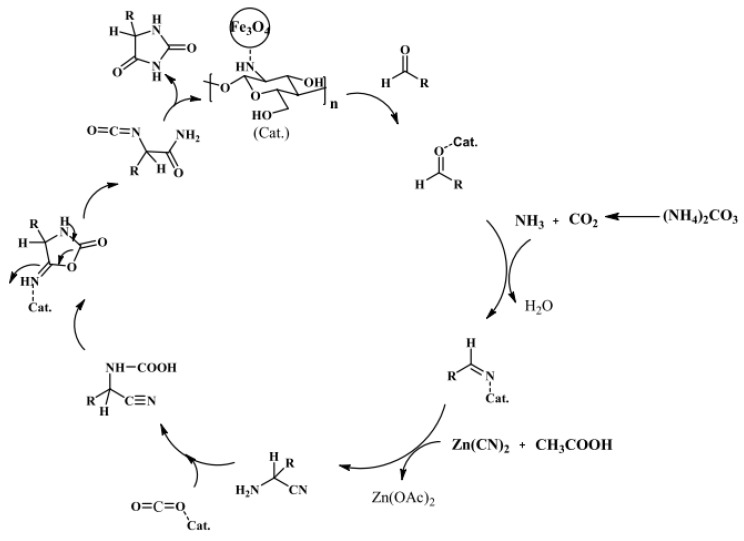
Plausible mechanism for the synthesis of 5-substituted hydantoins using Fe_3_O_4_-chitosan as a magnetic catalyst. Reaction conditions: aldehyde (1 mmol), ammonium carbonate (5 mmol), Zn(CN)_2_ (1 mmol), catalyst (20 mg), and 60 °C. Reprinted with permission from ref. [64]. Copyright 2016 Oxford International Collaboration Centre Press.

A chitosan shell on the surface of the MNP core can act as an efficient catalyst. Safari and Javadian proposed an easy method for the synthesis of 5-substituted hydantoins with high yields (80–97%) and short reaction times (12–25 min) by using a robust and magnet-recoverable catalyst, prepared via the simple adsorption of chitosan on the surface of Fe_3_O_4_ nanoparticles (Figure 14) [64].

**Figure 15:**
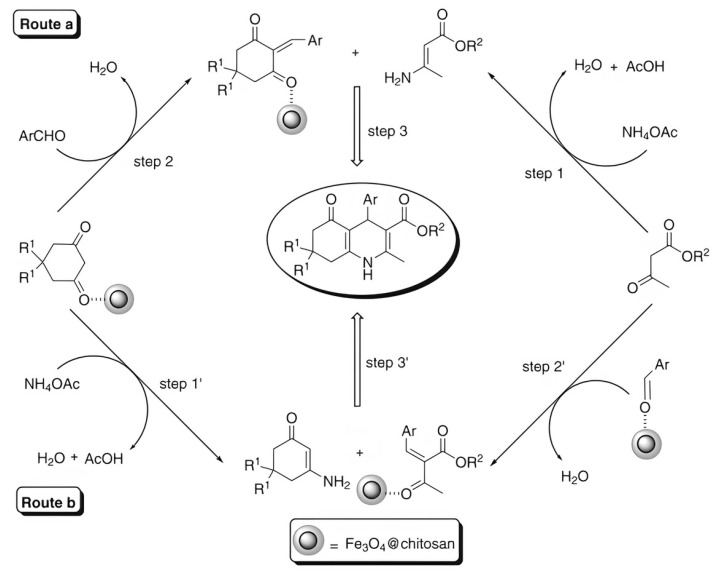
Two proposed mechanistic routes for the synthesis of 1,4-dihydropyridine derivatives using Fe_3_O_4_@chitosan as a catalyst. The first proposed mechanism: step 1; step 2; step 3. The second proposed mechanism: step 1′; step 2′; step 3′. Reprinted with permission from ref. [66]. Copyright 2016 Springer Nature.

In [66], a Fe_3_O_4_@chitosan nanocomposite was prepared by coating Fe_3_O_4_ nanoparticles with chitosan–glycolic acid gel; this was used as a green, magnetically recyclable, and efficient catalyst for the synthesis of chemically and biologically important dihydropyridine derivatives at room temperature under mild reaction conditions (Figure 15). This process allows for the easy and efficient synthesis of 1,4-dihydropyridine derivatives at high yields (87–95%). The catalyst is recovered via filtration and can be reused at least five times without a significant loss in catalytic activity.

**Figure 16:**
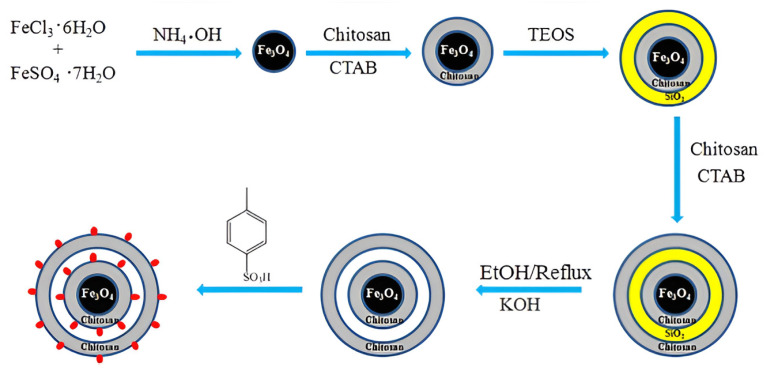
Schematic illustration of the synthesis of the mesoporous magnetic biomass-based acidic FCHC-SO_3_H catalyst [67].

Wang et al. [67] proposed an interesting way to prepare a magnetic chitosan acidic catalyst for the esterification of oleic acid with methanol. The synthesis of the catalyst includes the subsequent coating of Fe_3_O_4_ nanoparticles with chitosan or silica and chitosan and the further etching of the SiO_2_ middle layer from the triple shell. Finally, magnetic microspheres with a double chitosan shell structure were sulfonated to produce the Fe_3_O_4_@Chitosan-Hollow-Chitosan-SO_3_H (FCHC-SO_3_H) acidic catalyst (Figure 16). A novel FCHC-SO_3_H magnetic catalyst was found to show predominant catalytic performance in producing biodiesel. Under the optimum reaction conditions (a 15:1 methanol/oleic acid mole ratio and a 4 wt.% catalyst dosage at 80 °C for 3 h), a high biodiesel yield of 96.7% was obtained over FCHC-SO_3_H.

#### 2.3.2. Metal Catalysts on Magnetic Chitosan

The functionalization of MNPs with chitosan (CS) can provide a large number of hydroxyl, primary amino, and acetylamino groups on their surface. Furthermore, these groups can be converted easily to another coordinating ligand by using a chemical reaction with varieties of organic molecules, such as carbonyl compounds, esters, nitriles, etc. This affords a variety of metal complexes for various catalytic organic syntheses, such as coupling reactions, reductions, cycloadditions, and multicomponent reactions [65].

**Figure 17:**
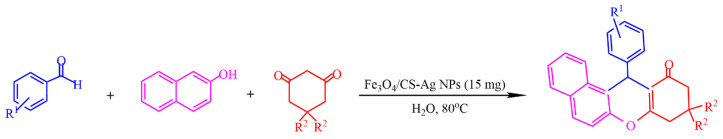
One-pot, three-component synthesis of 12-aryl-8,9,10,12-tetrahydrobenzo-[α]xanthene-11-one derivatives. Adapted from [68].

In [68], Fe_3_O_4_ nanoparticles were modified using –NH_2_ groups via the in situ coprecipitation of Fe^3+^ and Fe^2+^ ions in the presence of chitosan (CS) using NH_4_OH in an aqueous solution, resulting in a magnetic Fe_3_O_4_/CS composite. The adsorption of Ag^+^ ions on the surface of the latter, followed by the green reduction of Ag^+^ via ascorbic acid, yielded Fe_3_O_4_/CS-Ag NPs, which showed an excellent catalytic activity (85–96%) toward the one-pot, three-component synthesis of 12-aryl-8,9,10,12-tetrahydrobenzo[α]xanthene-11-one derivatives (Figure 17).

**Figure 18:**
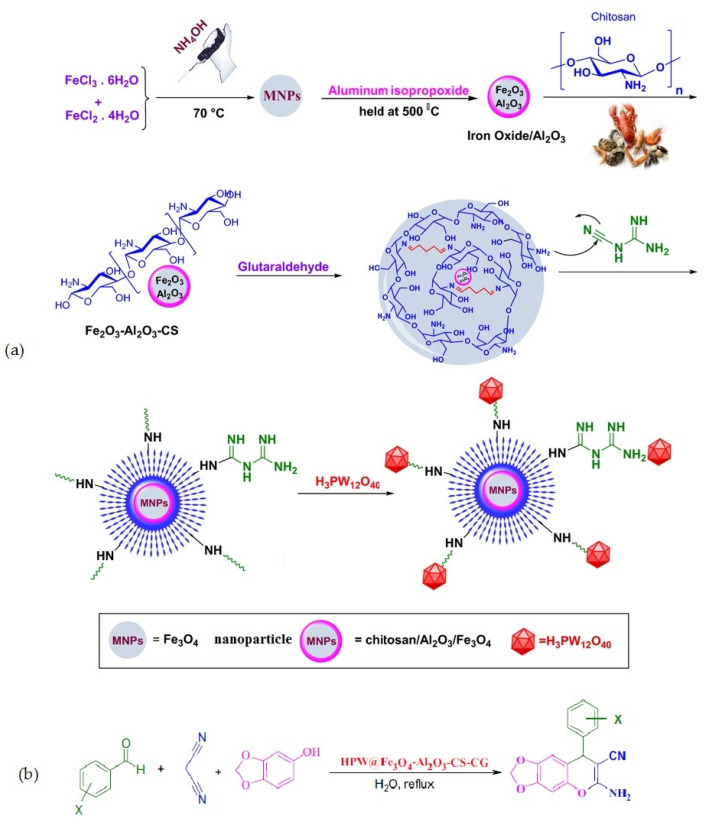
Preparation of HPA@Fe_3_O_4_-Al_2_O_3_/CS-CG (**a**) and its application in the synthesis of 6-amino-8-aryl-7-cyano-8*H*-[1,3] dioxolo-[4,5-g]-chromene (**b**). Reprinted from ref. [69].

Rahimzadeh et al. [69] used Al_2_O_3_/Fe_3_O_4_ nanoparticles coated with chitosan–cyanoguanidine as a support material to create a phosphotungstic acid catalyst. The catalytic activity of the fabricated composite (Figure 18a) was examined in a one-pot, three-component reaction involving diverse active methylene compounds, various aryl aldehydes, and malononitrile in water (Figure 18b). The results revealed that the composite showed efficient catalytic performance, and all reactions proceeded smoothly and led to the formation of the corresponding pyranochromene derivatives with high to excellent yields (80–97%).

**Figure 19:**
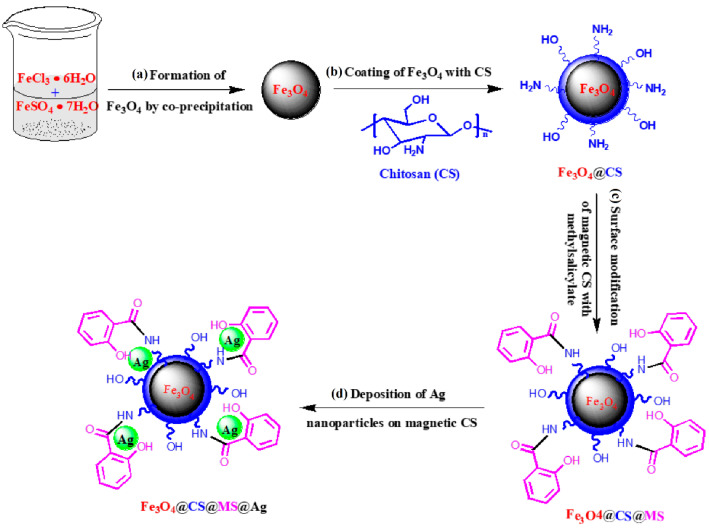
Synthetic procedure for the fabrication of Fe_3_O_4_@CS@MS@Ag as a catalyst. Reprinted from ref. [70].

In [70], a heterogeneous catalyst (denoted as Fe_3_O_4_@CS@MS@Ag) was fabricated using the deposition of silver nanoparticles on magnetic chitosan via an easy and facile modification of its surface using methyl salicylate (MS) (Figure 19). The catalyst (0.8 mg) showed excellent catalytic activity in the reduction of 4-nitrophenol (6 mL of 10.0 mg/L 4-NP aqueous solution) to 4-aminophenol in the presence of NaBH_4_ (1.0 mL of freshly prepared 1.0 mM NaBH_4_ aqueous solution) under ambient conditions. The catalyst was separated using an external magnet after each cycle of catalytic reaction and was reused effectively five times with almost 90% efficiency.

**Figure 20:**
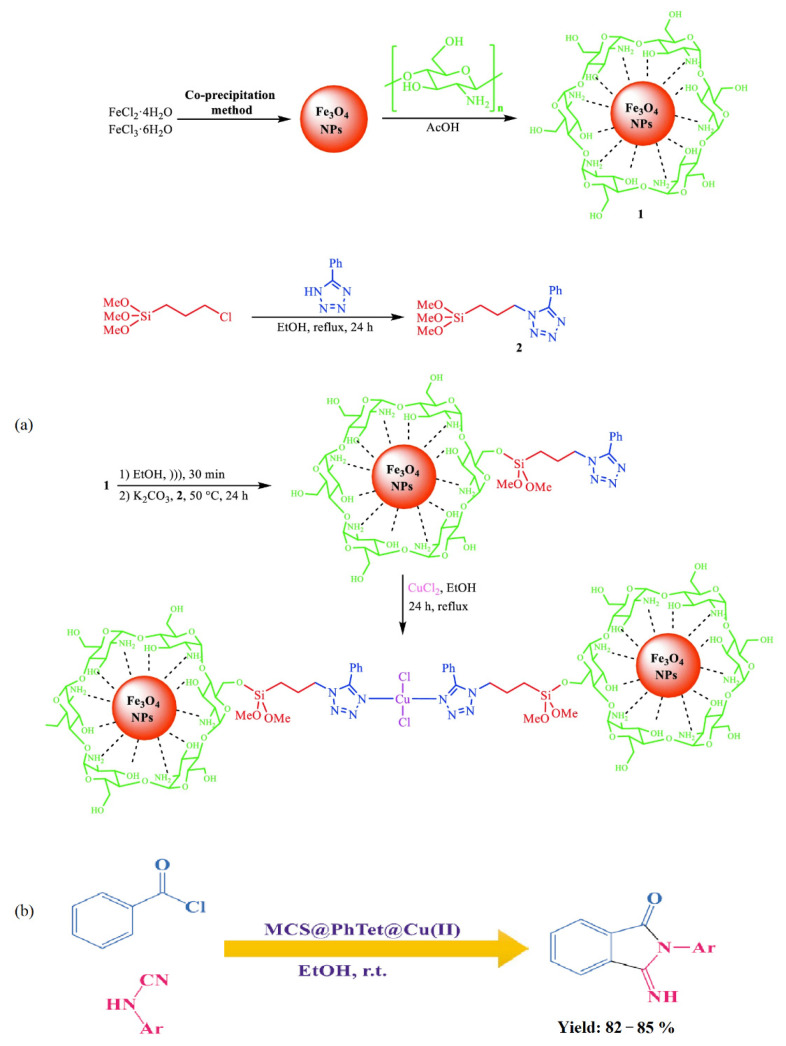
Preparation of MCS@PhTet@Cu(II) (**a**) and its application in the synthesis of 3-imino-2-phenylisoindolin-1-one derivatives (**b**). Reprinted from ref. [71].

Nasrollahzadeh et al. [71] synthesized a novel copper (II) complex of 5 phenyl 1H tetrazole immobilized on magnetic chitosan (MCS@PhTet@Cu(II)) (Figure 20a) as an effective catalyst. The catalytic activity of MCS@PhTet@Cu(II) was evaluated in the ultrasound-assisted synthesis of 3-imino-2-phenylisoindolin-1-one derivatives via the reaction between benzoyl chloride and arylcyanamides in ethanol under ambient temperature (Figure 20b). The catalyst showed high efficiency and recyclability even after five cycles with no significant loss of its efficiency.

#### 2.3.3. Enzymatic Catalysts on Magnetic Chitosan

**Figure 21:**
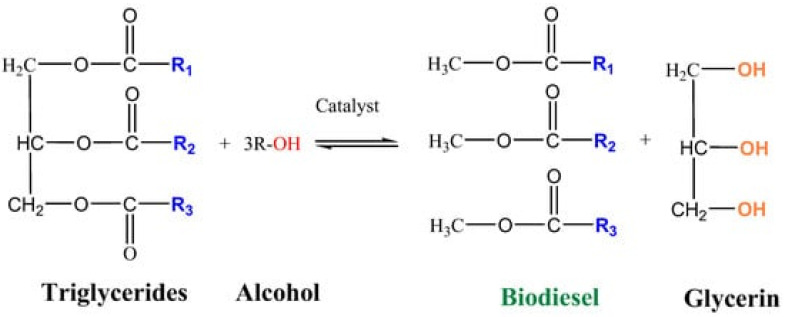
Transesterification reaction [73].

Chitosan-coated MNPs have become attractive as carriers for the immobilization of enzyme catalysts due to the ability of the polysaccharide to adsorb organic molecules. The immobilization of soluble enzymes on a chitosan shell can improve the stability of enzymes over a wide range of operating conditions [72]. On the other hand, the magnetic core makes it possible to recover these enzymatic catalysts from a reaction mixture for their fast and easy reuse. All of this can facilitate the widespread application of soluble enzymes in industrial processes. For example, such catalysts can be used in biofuel production via the transesterification of vegetable oils (Figure 21).

In [74], the enzymatic synthesis of ethyl esters from coconut oil (*Cocos nucifera*) and ethanol was evaluated in a bioreactor assisted by an electromagnetic field using lipase from Pseudomonas fluorescens immobilized on chitosan/magnetite beads. The attained results suggest that the synthesis of ethyl esters in this unconventional bioreactor assisted by a magnetic field was positively influenced by molar ratio and temperature, while magnetic induction was important for the establishment of the magnetically stabilized bed; in addition, this helped retain/separate the magnetic biocatalysts for further reuse. The transesterification reaction in the presence of the catalyst achieved a maximum conversion of around 12% under the optimal reaction parameters of 12 h, an oil/alcohol molar ratio of 1:11.25, 47.5 °C, and a magnetic induction of 9.7 mT.

Chen et al. [75] prepared magnetic whole-cell biocatalysts (MWCBs) by immobilizing Pseudomonas mendocina cells within Fe_3_O_4_–chitosan microspheres. The MWCBs were applied to the conversion of soybean oil into biodiesel, with a yield of 87.32% obtained under the optimum operating conditions of 48 h, a MWCB concentration of 10 wt.%, a water content of 10 wt.%, 35 °C, a methanol/oil molar ratio of 4:1, and a four-step addition of methanol. In addition, the MWCBs had excellent reusability and still produced a biodiesel yield of 83.57% after 10 cycles, which was higher than that of Fe_3_O_4_-uncontained whole-cell biocatalysts (74.06%).

Enzymatic catalysts immobilized on magnetic chitosan can also be used to degrade organic pollutants to CO_2_ and H_2_O for soil and wastewater treatment. In [76], cold-active crude enzymes from a psychrophile (xylene monooxygenase (XMO) and catechol 1,2-dioxygenase (C1,2D)) were immobilized on magnetic chitosan microparticles using glutaraldehyde as a linker. The optimization of the amount of each compound involved in immobilization (chitosan, glutaraldehyde, magnetic microparticles, and enzymes) showed that the highest immobilization yield was up to 98%. The immobilized enzymes showed improved pH tolerance ranging from 4.0 to 9.0, better temperature stability ranging from 5 °C to 50 °C, higher storage stability (~70% activity after 30 days of storage), and, more importantly, greater reusability (~40% activity after 10 repetitive cycles of usage) compared to their free form. Moreover, the immobilization of enzymes provided a two-fold increase in the p-xylene degradation rate in soil (10,000 mg/kg) and water (200 mg/L) samples.

**Figure 22:**
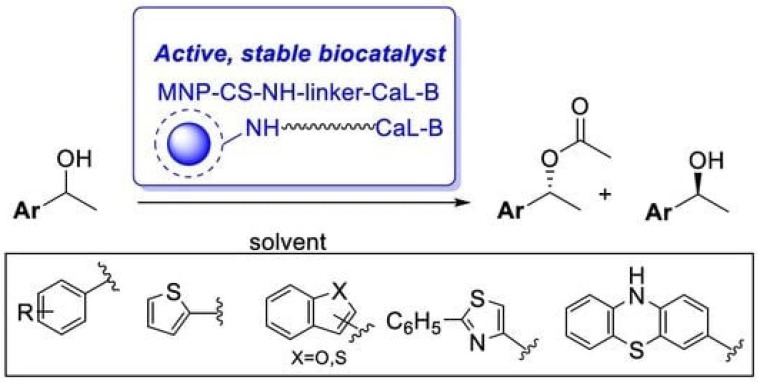
Enantiomer-selective transesterification of racemic ethanols mediated by immobilized CaL-B.

Another application of magnetic chitosan–enzyme catalysts is the synthesis of valuable chemicals or their precursors. For example, lipase B from Candida antarctica (CaL-B) immobilized by covalent binding on sebacoyl-activated chitosan-coated magnetic nanoparticles (MNPs) proved to be an efficient biocatalyst (49.2–50% conversion in 3–16 h and >96% enantiomeric excess) for the enzymatic kinetic resolution of some racemic heteroarylethanols via transesterification with vinyl acetate (Figure 22). Under optimal conditions (vinyl acetate, n-hexane, 45 °C), the biocatalyst remained active after 10 cycles [77].

**Figure 23:**
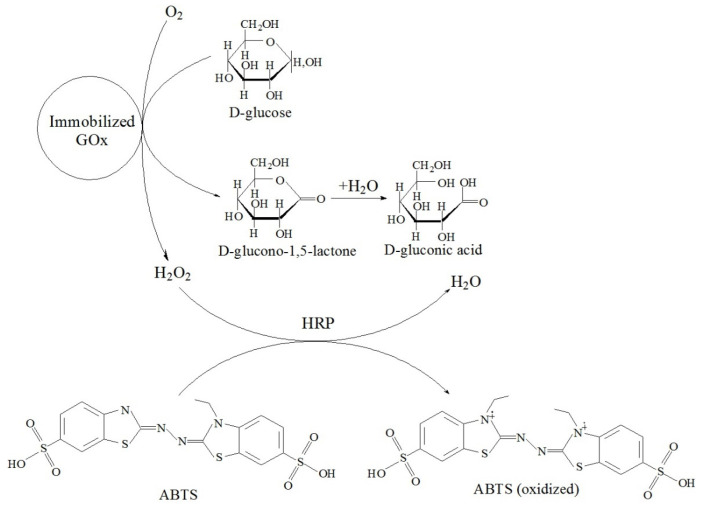
Schematic representation of *D*-glucose oxidation to *D*-gluconic acid in the presence of peroxidase (HRP) and 2,2′-azino-bis(3-ethylbenzothiazoline-6-sulfonic acid) diammonium salt (ABTS).

Tikhonov B B et al. [78] developed magnetically recoverable biocatalysts based on magnetite nanoparticles (MNAs) coated with an ultra-thin layer (about 0.9 nm) of chitosan (CS) ionically cross-linked with sodium tripolyphosphate (TPP). Excessive CS amounts were removed by multiple washings combined with magnetic separation. Glucose oxidase (GOx) was attached to the magnetic support via the interaction with *N*-hydroxysuccinimide (NHS) in the presence of carbodiimide (EDC), leading to a covalent amide bond. These steps resulted in the formation of the biocatalyst for *D*-glucose oxidation to *D*-gluconic acid (Figure 23), which can be used in the preparation of pharmaceuticals due to the benign character of the biocatalyst components. In order to choose the catalyst with the best catalytic performance, the amounts of all biocatalyst components were varied. The optimal biocatalyst—prepared using 0.1 g of CS, 0.05 g of TPP, 0.1 g of NHS, 0.04 g of EDC, and 0.01 g of GOx per 1 g of MNAs—showed 100% relative catalytic activity at pH 6 and 35 °C. The immobilization of GOx and the magnetic character of the support prevents GOx and biocatalyst loss and allows for repeated use.

### 2.4. Chitosan/Metal–Organic Framework Catalysts

In the past few years, metal–organic frameworks (MOFs) formed via the self-assembly of metal ions with organic ligands via strong coordination bonds have attracted massive attention. The major characteristic features of MOFs are their phenomenal surface area and porosity, high density of transition metals, and good chemical, mechanical, and thermal stability. These properties make MOFs perfect materials for catalytic application [79]. Among them, bio-based MOFs seem to be very promising due to the abundance, nontoxicity, and chemical properties of the renewable material sources (polysaccharides, amino acids, and peptides) used for their preparation [80]. Chitosan-based MOFs are no exception and can also be used as effective catalysts in various reactions.

Catalysts containing chitosan and MOFs can be used in wastewater treatment. Vigneswaran et al. reported [81] that a combination of a hetero-structured metal–organic framework of copper (II) benzene-1,3,5-tricarboxylate (Cu-BTC) with a Fe_2_O_3_ nano-photocatalyst using chitosan (CS) as a tethering agent resulted in the formation of a hybrid mesoporous nanocomposite (CS-Fe@Cu-BTC). The CS-Fe@Cu-BTC photocatalyst demonstrated 91% removal of paraquat within 60 min at pH 5. The durability of the CS-Fe@Cu-BTC nanocomposite was also established after four cycling processes.

**Figure 24:**
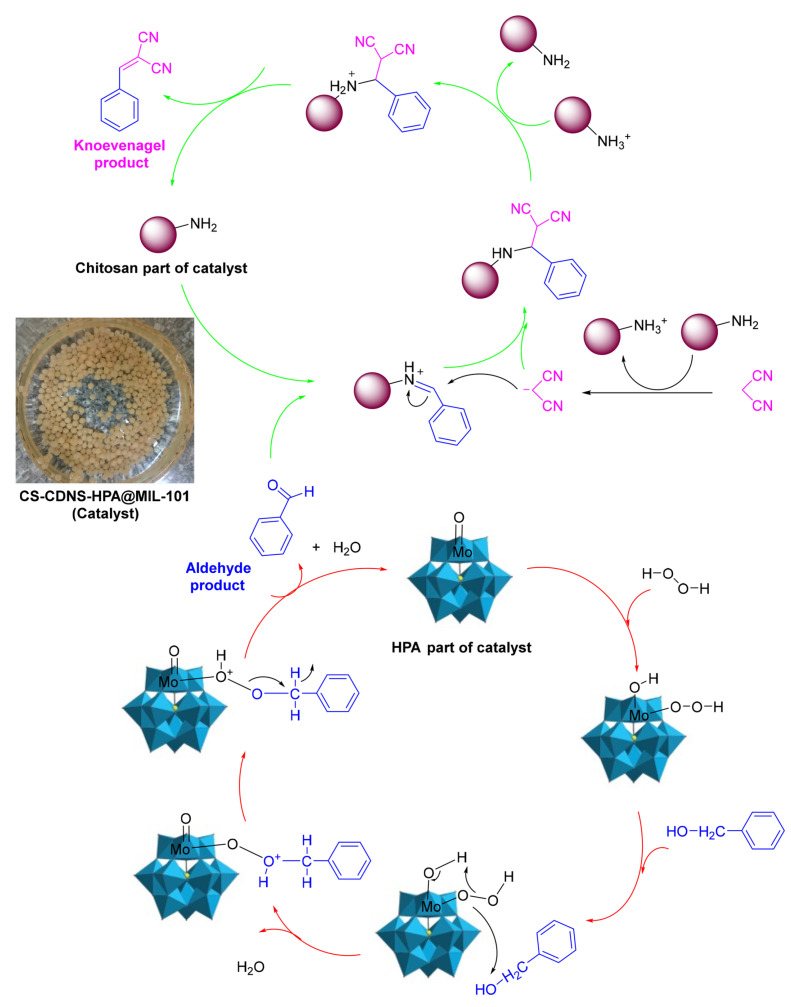
The plausible mechanism for the cascade alcohol oxidation–Knoevenagel condensation reaction, highlighting the catalyst effect. Reprinted from ref. [82].

Another application of chitosan/MOF catalysts is tandem reactions, in which bonds are developed without isolating the intermediates by altering the reaction conditions or through the inclusion of chemical reagents [80]. Sadjadi et al. [82] described the synthesis of a novel bi-functional catalytic composite, in which a MIL-101(Fe) metal–organic framework modified with phosphomolybdic acid (HPA) and cyclodextrin-based nanosponges (CDNS) was incorporated into chitosan (CS) beads. CS-CDNS-HPA@MIL-101 was examined in alcohol oxidation and the cascade alcohol oxidation–Knoevenagel condensation reaction. It was found that the designed catalyst possessed both an acidic feature and redox potential, which could promote both reactions in aqueous media at 55 °C, and various substrates with different electronic features could tolerate the aforementioned reactions to furnish the products, with a 75–95% yield. That is, the amino groups on the chitosan served as catalysts for the Knoevenagel condensation reaction, and phosphomolybdic acid (HPA) catalyzed the oxidation process (Figure 24). The cyclodextrin nanosponge mainly acted as a phase transfer agent. The catalyst could be readily recovered and recycled for five runs with a slight loss in catalytic activity. Moreover, the measurement of phosphomolybdic acid leaching showed that its incorporation in a metal–organic framework and bead structure could suppress its leaching, which is considered a drawback for this compound.

Chitosan/MOF composites can also be used as a support material for metal catalysts. Wang et al. [83] discussed the preparation of chitosan (CS) microspheres coated with a ZIF-8 metal–organic framework using polydopamine (PDA) as a connecting material. A CS/PDA@ZIF-8 core–shell composite, which possessed regular morphology, uniform size, and a large surface area (353.1 m^2^/g), was used as a support material for the immobilization and stabilization of palladium nanoparticles (NPs). The CS/PDA@ZIF-8@Pd catalyst performed well in an array of reactions at room temperature. For example, the Kapp value of the p-nitrophenol reduction reaction reached 0.0426 s^−1^, and the TOF of the Suzuki coupling reaction reached 128 h^−1^ (99% yield of biphenyl products). The prepared catalyst demonstrated a reusability of six cycles in the Suzuki reaction with a slight loss in catalytic activity.

Thus, the chemical properties of chitosan make it possible to obtain effective catalysts for various organic transformations (coupling reactions, reduction of nitroaromatics, transesterification, hydrogenation, oxidation, multicomponent reactions, cascade reactions, decomposition of organic molecules, etc.) using different approaches. The simplest one is based on the binding affinity of the –NH_2_ and –OH functional groups of the polysaccharide toward transition metal ions. The ability of chitosan macromolecules, when dissolved in an acidic medium, to be precipitated at pH > 6.3 is used in the preparation of catalytic microspheres, where chitosan serves as a support for metal catalysts. This ability is also used to functionalize inorganic materials (alumina, silica, magnetite, etc.) and obtain core–shell composites, in which the chitosan shell can act as both a catalyst and a linker to immobilize metal or nonmetal catalysts (enzymes and sulfonic acids) on their surface. The cross-linking of the polysaccharide macromolecules with aldehydes is used to improve the mechanical strength of chitosan microspheres, promote the strong fixation of the chitosan shell to the surface of an inorganic support, and also reduce the probability of metal NP leaching. Chitosan can also be modified to form chelate ligands (Schiff bases, etc.) with better affinity for transition metal ions, which also affects their behavior in catalytic processes. Of particular interest is the combination of chitosan with organometallic frameworks (MOFs), where the biopolymer can act as a binding agent between MOFs and active particles or as a support for MOF-based catalysts. Studies on the immobilization of soluble enzymes on magnetic chitosan, which can allow for the widespread application of enzymatic catalysts in industrial processes, also show promise.

## 3. Pectin-Based Nanocomposites

**Figure 25:**
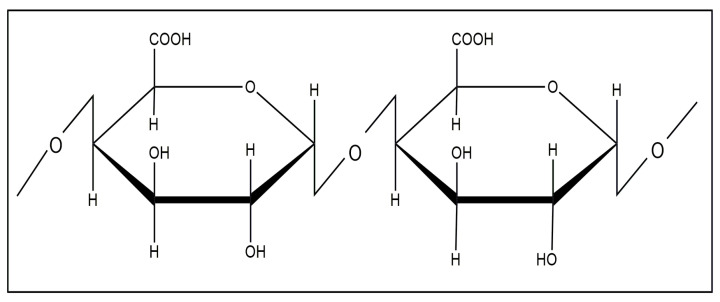
Structural formula of pectin.

Pectins are heteropolysaccharides that are widely used in the medical, food, and cosmetic industries due to their emulsifying, gelling, thickening, and stabilizing properties [84,85]. The structure of pectin mainly consists of *D*-galacturonic acid residues connected through an α-1,4-glycosidic bond to a polymer chain (Figure 25).

The presence of a large number of functional groups in the structure provides unique properties and a wide range of pectin applications [84,85,86,87]. Pectin is considered a safe, non-toxic, inexpensive product to produce and represents an easily accessible polysaccharide [88,89].

Pectins are separated by the degree of methoxylation—the ratio of the number of methoxyl groups (O-CH_3_) to all the acid residues in the molecule. According to the degree of esterification, all industrial pectin species are classified as high methoxyl (with an esterification degree that is equal to or more than 50%) or low methoxyl (with an esterification degree of less than 50%) [90]. The degree of esterification is an important parameter that determines the special properties and functionality of pectin (stability, viscosity, gelation, strength, etc.) [91,92,93,94,95].

Pectins are contained in plant cell walls and are abundant in plant waste [96,97]. Their composition and structure strongly depend on the source of pectin, extraction stages, conditions, etc. [96]. On an industrial scale, about 85% of pectin is extracted from citrus peels and apple pomace, which are characterized by a high pectin content (18–30%) and are accessible as agricultural waste [88]. Sugar beet pulp, sunflower seeds, mangoes, bananas, grapefruit, etc., are considered alternative sources of pectin [90,98,99].

Traditional methods of pectin extraction involve the use of hot water (60–100 °C) in an acidic medium for several hours [96,100,101]. In recent years, various alternative methods, such as enzymatic [102], microwave [96,103], and ultrasonic [102,103,104,105] methods, have been used to increase the yield of pectin, which can improve process efficiency and are more environmentally friendly and cost-effective [97,101,104].

### 3.1. Pectin and Pectin–Inorganic Composites as Catalysts

**Figure 26:**
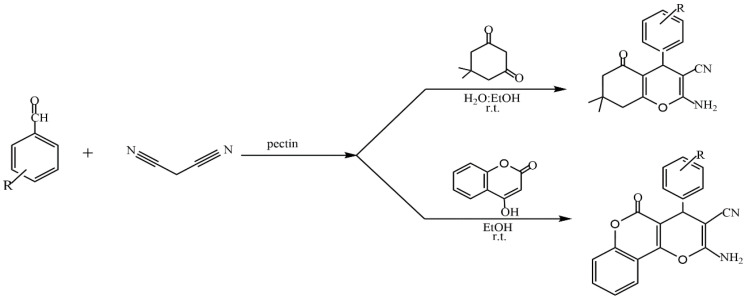
Synthesis of tetrahydrobenzo[b]pyran and 3,4-dihydropyrano[c]chromene derivatives in the presence of pectin.

The presence of a large number of carboxyl and hydroxyl groups in the molecules allows pectins to be used to design various materials such as sorbents for wastewater treatment [106,107], organic–inorganic hybrid composites [108,109,110,111], and catalysts [24]. The study of the catalytic properties of pectin and pectin-containing nanocomposites has been described in [112,113,114,115,116,117,118,119,120,121]. In several reviews [24,36,112,114], the authors discuss the prospect of applying the most common polysaccharides, including pectin, to catalytic processes (oxidation, Heck, Suzuki–Miyaura, cross-coupling reactions, etc.). Kangani et al. reported the green synthesis of tetrahydrobenzo[b]pyran and pyrano[c]chromene derivatives (Figure 26) in the presence of natural pectin in water–ethanol and ethanol media at room temperature [112].

**Figure 27:**
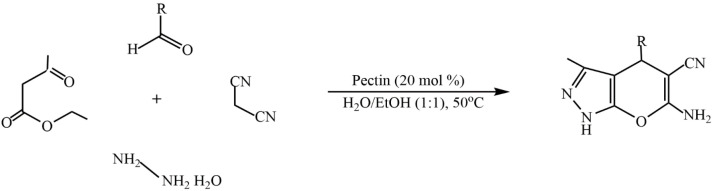
Synthesis of dihydropyrano[2,3-c]pyrazole derivatives.

The advantages of such types of synthesis are high product yields, short reaction times, mild synthesis conditions, and the use of natural, easily accessible, and inexpensive pectin as a catalyst. The catalytic properties of pectin are comparable to those of other catalysts. The environmentally friendly synthesis of biologically valuable heterocyclic compounds (dihydropyrano[2,3-c]pyrazole derivatives) by using the one-pot method is proposed in [113]. In this study, pectin was used as a biopolymer catalyst to rapidly access dihydropyrano[2,3-c]pyrazole derivatives in an aqueous ethanol medium over a short period of time. Comparative studies on the catalytic properties of the developed catalyst using known catalytic systems for the synthesis of dihydropyrano[2,3-c]pyrazole derivatives were carried out (Figure 27).

The results showed that pectin is an effective green catalyst for the production of biologically active compounds. Acosta et al. used the cation-binding capacity of pectin to reduce the amount of calcium ions leached into biodiesel [119]. The main disadvantage of biodiesel production via the reaction of vegetable oil transesterification using an efficient CaO catalyst is the presence of a large amount of calcium ions in the obtained biodiesel. The calcium content exceeds the permissible concentration (ASTM D6751) [119,122]. In this work, a CaO-based catalyst was prepared using a precipitation method with Na_2_CO_3_ and Ca(NO_3_)_2_ in the presence of pectin, followed by calcination. The most active catalyst, CaP-600, was prepared at a temperature of 600 °C. The synthesized catalysts showed high activity in the reaction of soybean oil transesterification to obtain biodiesel.

The design of pectin–inorganic composites for catalysis applications is of great interest [115,116,117,118]. When designing this type of material, the combination of the advantages of both components determines the specific properties of these nanocomposites. The strong synergetic interaction between them leads to the formation of composites with desired properties that positively affect the physicochemical properties of nanocomposite materials.

It should be noted that the proposed syntheses of pectin-based catalysts are generally considered environmentally friendly. Attallah et al. developed a pectin/chitosan/zinc oxide (Pec/CS/ZnO) nanocomposite for the removal of carbamazepine (CBZ) from aqueous solutions under direct sunlight [115]. In the organo-inorganic composite, chitosan and pectin act as the adsorbent for carbamazepine. Zinc oxide is responsible for the photocatalytic degradation of CBZ. The addition of zinc oxide to pectin and chitosan, according to the authors, will bring new and unique functional properties and increase the efficiency of the synthesized nanocomposites in carbamazepine reduction. The optimal conditions for CBZ reduction were 0.5 g/L Pec/CS/ZnO nanocomposite, pH 4, and an experimental time of 3 h. Under these conditions, the degradation efficiency of 10 mg/L CBZ was 69.5%, with a rate constant (k) of 0.00737 min^−1^ and a half-life of 94 min. Pectin has the ability to improve the dispersion of the developed material and also acts as a stabilizer of nanoparticles (NPs) during their synthesis [116].

Nazeri and coworkers used pectin as a support for the preparation of a heterogeneous catalyst based on metal phthalocyanines [118]. Before the immobilization of copper tetra-aminophthalocyanine, pectin was oxidized with periodate. The catalytic activity of the developed catalysts was investigated in the reaction of CO_2_ fixation to obtain cyclic carbonates.

**Figure 28:**
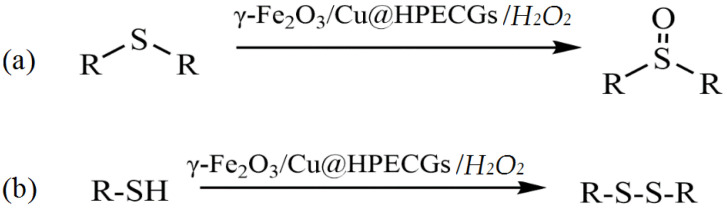
Chemoselectivity in the oxidation of sulfides and the oxidative coupling of thiols (**a**), as well as the oxidative coupling of thiols by using γ-Fe_2_O_3_/Cu@HPECGs with H_2_O_2_ (**b**).

The development of catalytic systems based on non-platinum metals is the most reasonable compared to that of catalysts based on noble metals. Yarmohammadi et al. developed a method for obtaining iron and copper nanoparticles embedded in a pectin hydrogel matrix [123]. The pectin hydrogel was prepared in the presence of calcium chloride by using a physical cross-linking method. Then, iron and copper nanoparticles were deposited on the surface of the hydrogel matrix. The synthesized composite demonstrated high activity, selectivity, and stability in the oxidative coupling of thiols with disulfides in the presence of the green oxidant hydrogen peroxide (Figure 28).

Thus, the preparation of polymer–inorganic composites by modifying the surface of inorganic materials with a polymer has a number of advantages, which include the improvement of their catalytic properties, multifunctionality, the widening of application possibilities for inorganic materials (metals, oxides, and sorbents), etc. It should be noted that pectin itself can act as a catalyst and a binding agent for metals (e.g., calcium ions in biodiesel production).

### 3.2. Metal Nanoparticles Stabilized with Pectin

In recent years, polysaccharides have been used to stabilize, microencapsulate, and modify the active phase of the nanoparticles (NPs) in catalytic systems. There are some works devoted to the preparation and application of pectin-stabilized metal nanoparticles in catalysis.

The stabilizing role of pectin was confirmed by Baran [120]. The agar/pectin composite (APC), which is characterized by high thermal stability, high active surface area, and strong covalent bonds, was used as a stabilizer of palladium nanoparticles. No toxic reducing agents were used to immobilize the nanoparticles on the composite surface. SEM micrographs showed that the palladium nanoparticles had a spherical morphology and were homogeneously distributed on the APC surface; the average size of the PdNPs was 34–54 nm. The synthesized catalysts showed high efficiency in Suzuki–Miyaura cross-coupling reactions and the reduction of o-nitroaniline at room temperature in an aqueous medium. The results indicate that biopolymer-containing composites can be used as stabilizers for nanoparticles of various noble metals. Thus, Baran T developed a promising support for the fixation of metal ions—pectin modified with a Schiff base (Pct) [124]. The obtained material has a good adsorption capacity in relation to Pd ions. The catalytic properties of the synthesized Pct-Pd catalyst were investigated in the Suzuki–Miyaura reaction. High conversion and reproducibility values were obtained. The conversion reached 91% after 12 consecutive portions of the substrate, and no significant leaching of Pd ions from the surface of the modified support was observed. The synthesized pectin-based catalyst is promising for industrial application.

In [117], an environmentally friendly method for the preparation of pectin-stabilized palladium nanoparticles obtained by exposing an aqueous solution of PdCl_2_ (100 mL, 1 mM) to pectin without an additional reducing agent was presented. The synthesized nanoparticles were investigated in the Mizoroki–Heck reaction between various aryl halides and n-butyl acrylate under solvent-free conditions. The catalyst can be reused for six cycles without a significant loss of catalytic activity.

A method for obtaining Pt, Pd, Au, and Ag nanoparticles by reducing their ions in pectin in the absence of additional reducing agents was shown in [125]. Their electrocatalytic activity was investigated in the electro-oxidation of glucose. Ponce et al. showed the promising application of catalysts prepared in the presence of pectin in the production of biodiesel (biodiesel yield was about 95%) [126]. The disadvantage of the synthesis was the use of high-temperature calcination stages (600 °C).

**Figure 29:**
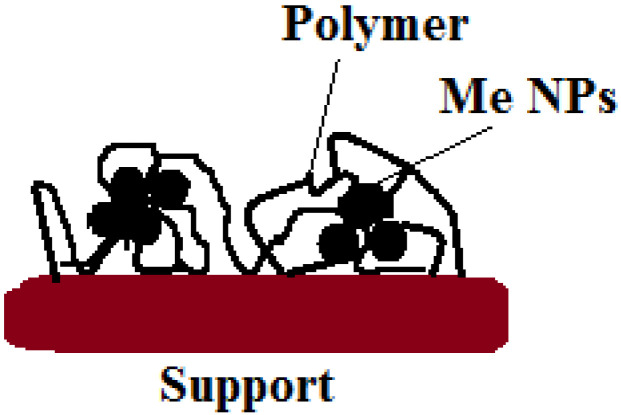
Scheme of the formation of a polymer-stabilized palladium catalyst deposited on a solid support for the process of acetylene compound hydrogenation.

In the development of heterogeneous catalysts, the leaching of the active phase is one of the most challenging tasks. In order to solve this problem, various approaches are used. A simple method for obtaining pectin- and PEG-stabilized palladium catalysts deposited on a solid support was proposed by Zharmagambetova A et al. [127]. The synthesis of catalysts involves the low-temperature stages of the sequential deposition of polymer and metal ions (Pd, Pt, etc.) on inorganic supports. The catalysts have shown activity, selectivity, and stability in the hydrogenation of n-hexyn-2 and phenylacetylene. It should be noted that the catalytic properties (activity and selectivity) of the polysaccharide-based catalyst were comparable to those of the catalysts based on synthetic polymers. The same approach to the synthesis of catalysts was used in [22,23,128]. In all cases, the modification of a traditional support material with polymers led to an increase in its affinity for transition metal ions. Moreover, the modification with polymers provides the formation of stabilized metal nanoparticles (NPs) on the surface of support materials (Figure 29). The polymer “layer” promotes the uniform distribution of the active phase (metal NPs) and prevents its agglomeration, while the inorganic support provides the easy separation of the catalyst from the reaction products and resistance to the action of the solvent. The developed method allows for an increase in the efficiency of catalysts in low-temperature hydrogenation processes and for carrying out the process under the atmospheric pressure of hydrogen.

**Figure 30:**
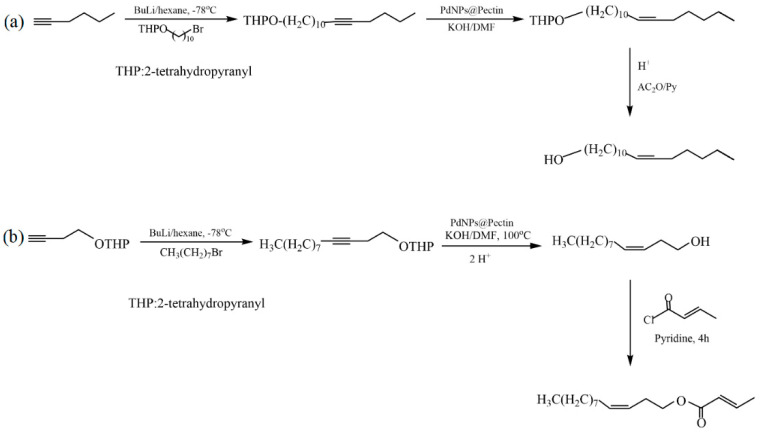
Synthesis of Plutella xylostella (**a**) and Cylas formicarius (**b**) pheromones.

Le et al. proposed an in situ synthesis of palladium nanoparticles (PdNPs) using pectin extracted from the leaves of Cyclea barbata (a species of flowering plant) as a green reducing and stabilizing reagent [33]. The developed PdNP@pectin catalyst showed high catalytic activity in the Heck reaction in an aqueous medium. The synthesized catalysts were investigated in the hydrogenation of alkynes to cis-alkenes and in the synthesis of the sex pheromones of Plutella xylostella ((*Z*)-11-hexadecen-1-yl acetate) and Cylas formicarius ((*Z*)-3-dodecen-1-yl(*E*)-2-butenoate) (Figure 30). The yields of (*Z*)-11-hexadecen-1-yl acetate and (*Z*)-3-dodecen-1-yl(*E*)-2-butenoate were 70% and 68%, respectively.

Thus, in the formation of catalytic systems based on metal nanoparticles, pectin is a potential stabilizing agent that prevents the leaching and aggregation of the active phase of nanoparticles.

### 3.3. Pectin-Based Magnetic Catalysts

Magnetic catalysts have attracted much attention because of their ability to be separated from the reaction mixture using a magnetic field, which is a more efficient, less energy-consuming, and faster way to separate small particles compared to filtration and centrifugation [129]. Polysaccharides containing functional groups can be used for modifying magnetic nanoparticles [121,130].

**Figure 31:**
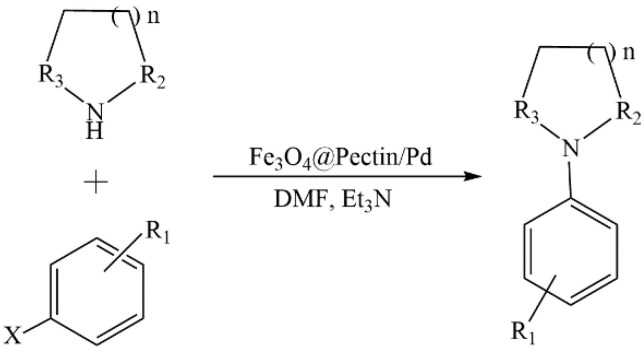
Buchwald–Hartwig cross-coupling reaction.

Li et al. synthesized new magnetic Au nanoparticles modified with pectin (Fe_3_O_4_/pectin/Au) [121]. The developed composite was investigated in the catalytic reduction of nitroarenes in a water–ethanol medium without any promoters or ligands. Due to its strong paramagnetism, the catalyst was easily recovered and reused for 11 cycles without a significant loss of reactivity. Similar composites based on pectin and Fe_3_O_4_ were used to synthesize a Pd nanocatalyst (Fe_3_O_4_@pectin/Pd) [130]. The catalyst showed high reactivity in the C-C and C-N cross-coupling reactions of Suzuki and Buchwald–Hartwig reactions, respectively (Figure 31). The catalyst was easily extracted using an external magnet and then reused. The slight leaching of palladium was observed over several tests.

Ghamari Kargar et al. proposed an approach for the development of a magnetic catalyst based on pectin (Fe_3_O_4_@PectinImidazole SO_3_H-Cu(II)) for the oxidation of benzyl alcohols to aldehydes [131]. The advantages of this method are low catalyst loading, obtaining pure products, using inexpensive metals, the absence of solvents, using a biodegradable and environmentally friendly support, and the stability of the catalyst.

**Figure 32:**
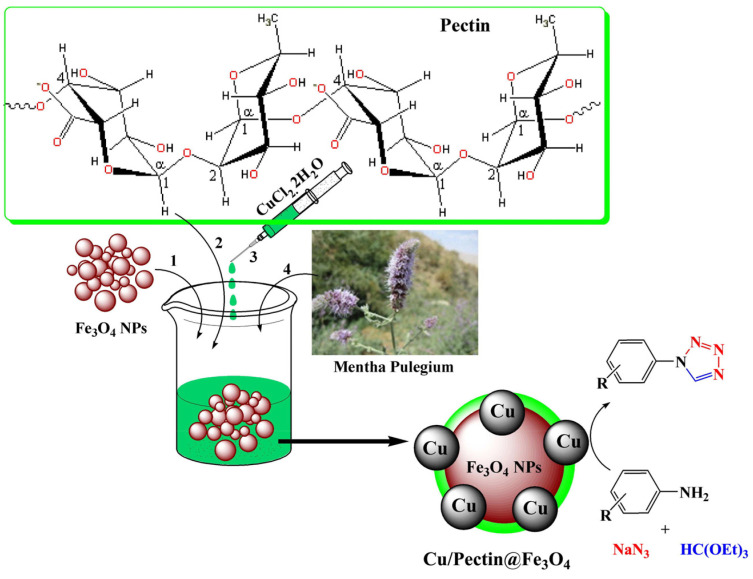
Preparation of a Cu/Pectin@Fe_3_O_4_ nanocomposite and its application for the synthesis of 1-substituted-1H tetrazoles. Reprinted from ref. [132].

Xue et al. developed a Cu/pectin@Fe_3_O_4_ nanocomposite (Figure 32) [132]. The synthesized nanocomposite was thoroughly characterized using a wide range of physicochemical methods such as FT-IR, FESEM, TEM, and ED. The catalyst was investigated in the solvent-free synthesis of variously substituted 1H-tetrazoles. The nanocomposite also showed biological activity.

Khashei Siuki et al. tested a magnetic catalyst based on pectin and nickel (Fe_3_O_4_@Pectin@Ni (II)) in the oxidation reaction of alcohols [133]. The developed catalysts showed high stability. The simplification and cost reduction of the preparation of xanthine analogs were achieved.

**Figure 33:**
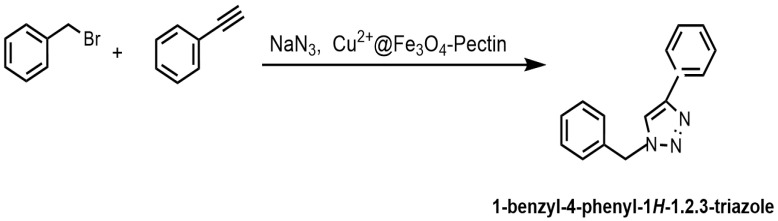
Preparation of Cu^2+^@Fe_3_O_4_–pectin and its application in regioselective three-component triazole synthesis.

Doustkhah et al. reported that Fe_3_O_4_ nanoparticles encapsulated in apple pectin (Fe_3_O_4_–pectin) can be used as a magnetic support material for a copper catalyst [134]. The developed Cu^2+^@Fe_3_O_4_–pectin nanocomposite was prepared via the coprecipitation of Fe(II/(III) ions in an alkaline solution, followed by the adsorption of copper(II) ions (Figure 33). The formation of magnetic nanoparticles in the pectin network was established. Pectin also served as a chelating agent to fix copper ions. The developed catalyst was then used in regioselective three-component triazole synthesis (Figure 33); the reaction conditions were benzyl bromide—1 mmol; NaN_3_—1.2 mmol; phenylacetylene—1 mmol; H_2_O—1 mL; and stirring the reaction for 4 h at room temperature.

Thus, magnetic catalysts based on pectin are characterized by improved catalytic properties, low catalyst loading, lower costs, and a biodegradable component used in the synthesis.

## 4. Cellulose-Based Nanocomposites

**Figure 34:**
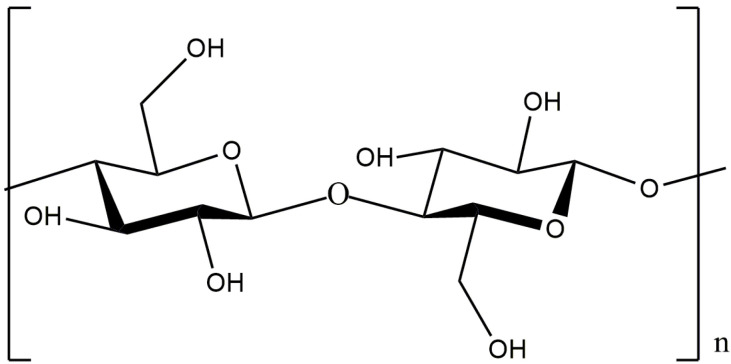
Structural formula of cellulose.

Cellulose is one of the most widespread natural polysaccharides. Natural cellulose is a linear polymer consisting of consecutive β-*D*-glucopyranose links and is characterized by a large number of OH- groups in the polymer chain, which determines the excellent chemical activity of cellulose (Figure 34).

Due to its unique characteristics (high biocompatibility, biodegradability, low toxicity, and ability for chemical modification), cellulose is actively used in the food, medicine, construction, and textile industries [135,136]. Many studies have been devoted to the development and application of cellulose-based catalysts [135,136,137,138,139,140,141,142,143].

### 4.1. Cellulose as a Catalyst Support and Stabilizing Agent

Due to its unique architectural structure, cellulose is considered an ideal support for fixing nanoparticles and stabilizing them for the creation of nanocatalysts.

Li et al. provided a description of various cellulose-based nanocatalysts prepared using the layer-by-layer (LbL) self-assembly method [135]. The synthesized nanocatalysts are three-dimensional porous structures with a high specific surface area. They demonstrated strong catalytic properties in photocatalysis processes (photodegradation of organic dyes and photocatalytic hydrogen production).

**Figure 35:**
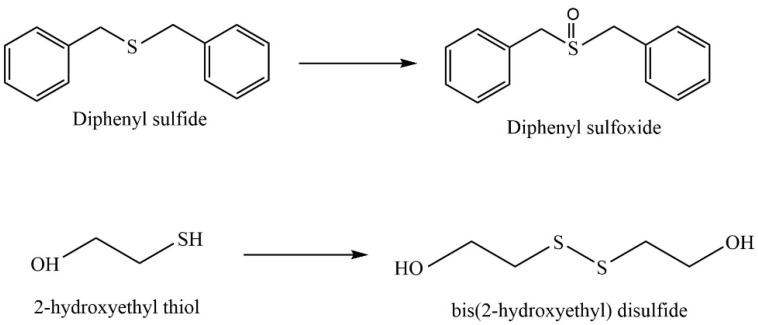
Oxidation of sulfides and thiols.

Naeimi et al. [137] synthesized a nanocatalyst based on bentonite clay (natural bentonite), cellulose extracted from wheat waste, and a vanadium complex for use as a catalyst in the oxidation of sulfides and thiols (Figure 35); the sulfide oxidation conditions were Cel/Ben/V nanocomposite—20 mg; 30% H_2_O_2_—0.33 mL; sulfide—1 mmol; EtOH—2 mL; T—25 °C; and magnetically stirred for 2 h or under ultrasonic irradiation within 1 h; the thiol oxidation conditions were Cel/Ben/V nanocomposite—20 mg; 30% thiol—1 mmol; CH_3_CN—2 mL; T—25 °C; and magnetically stirred for 2 h or under ultrasonic irradiation within 1 h.

**Figure 36:**
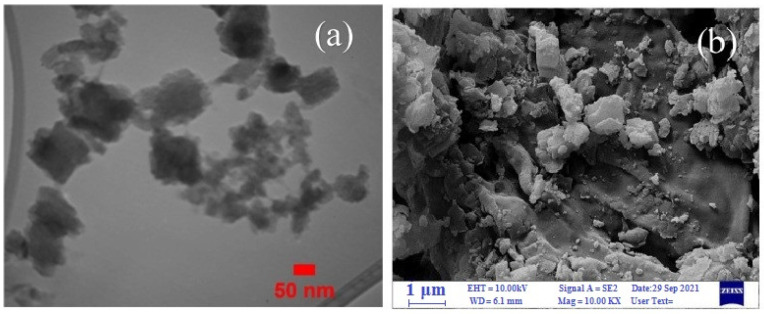
TEM (**a**) and SEM images (**b**) of Cel/Ben/V. Reprinted from ref. [137].

The developed catalysts showed high efficiency in the oxidation of various substrates. During the preparation of the composites, ultrasonic treatment was used instead of the usual mixing. A reduction in composite preparation time was achieved, and a high yield of reaction products was obtained. TEM images of the Cel/Ben/V nanocatalyst indicated the formation of 18–105.5 nm particles (Figure 36a). The SEM image clearly showed the morphology of the nanocatalyst (Figure 36b); the polymer matrix, bentonite, and the vanadium complex were detected.

**Figure 37:**
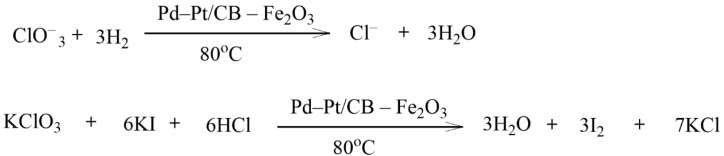
Hydrogenation of chlorate on a Pd-Pt/CB-Fe_2_O_3_ support.

Sikora et al. used CB as a support for the synthesis of Pd and Pt catalysts for the catalytic hydrogenation of chlorate (Figure 37) [139]. The advantage of this study was the low content of noble metals (<0.6 wt.%) in the catalyst. Fe_2_O_3_ was used as the catalyst promoter. The degree of substrate transformation reached 92.5%. The continuous hydrogenation of chlorate in the Pd-Pt/CB-Fe_2_O_3_ catalytic system was carried out. The developed catalyst showed good results. Over 160 min in the presence of this catalyst, the chlorate content in the reaction mixture reached a zero value.

**Figure 38:**
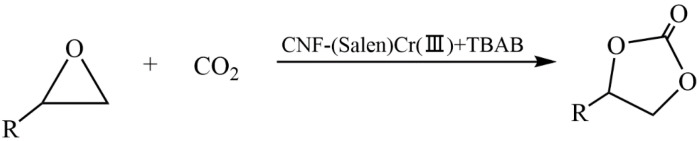
Synthesis of cyclic carbonate using various epoxides.

Liu et al. developed a new heterogeneous catalyst based on cellulose nanofibers (CNF) and a (Salen)Cr(III) complex to produce cyclic carbonates via a CO_2_ cycloaddition reaction with epoxides (Figure 38) [140]. The method of preparing the catalyst included the modification of CNFs with (3-aminopropyl)triethoxysilane (APTES), the synthesis of a Schiff base with salicylaldehyde, and a subsequent treatment with chromium chloride. The developed heterogeneous catalyst CNF-(Salen)Cr(III) showed high efficiency in the cycloaddition reaction of CO_2_ to epichlorohydrin under mild conditions with the participation of the co-catalyst TBAB. The yield of chloropropene carbonate was 97.8%. The catalyst based on cellulose nanofibers (CNFs) and the (Salen)Cr(III) complex retained its catalytic activity after recycling tests.

Gia-Thien Ho et al. proposed an environmentally friendly method to obtain silver nanoparticles on cellulose aerogel (CA) extracted from water hyacinth for the hydrogenation of nitrophenol in the presence of NaBH_4_ in an aqueous medium [141]. The obtained results showed that at room temperature, in the presence of a 1.5Ag/CA2.0 catalyst, the reaction was completed within 10 min. After five recycling tests, the catalyst did not lose its activity.

El Idrissi et al. synthesized a heterogeneous cobalt catalyst supported on a TEMPO–cellulose aerogel [142]. The catalyst preparation method involved the reduction of cobalt sulfate with NaBH_4_ in the presence of an aqueous suspension of TEMPO–cellulose at room temperature. TEMPO–cellulose prevents the connection of cobalt ions with boron. The results of the catalyst physicochemical study (SEM, XRD, EDX, et al.) confirmed the formation of cobalt nanoparticles uniformly distributed on the aerogel. The synthesized catalyst showed high efficiency in the reduction reactions of 4-nitroaniline, 4-nitrophenol, and 2-nitrophenol in water in the presence of sodium borohydride.

**Figure 39:**
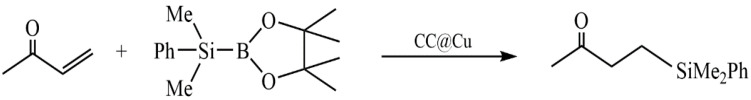
Reaction of α,β-unsaturated compounds in the presence of a catalyst.

Zhang et al. developed a green heterogeneous copper catalyst based on a chitosan/cellulose composite using a cross-linking method [143]. The developed catalyst was tested in the silylation reaction of α,β-unsaturated compounds in an aqueous medium (Figure 39). The advantages of the proposed technology include no need for bases, ease of use, availability, and the reusability of the catalyst.

In [144], the simple synthesis of palladium nanoparticles fixed to nanoporous microspheres of cellulose was proposed. The obtained catalyst was tested in the Suzuki–Miyaura coupling reaction and showed high catalytic activity and stability. The TOF was 2126 h^−1^. The catalyst remained active for six continuous cycles.

Shi et al. used cellulose fibers as templates for the synthesis of copper catalysts based on zinc oxide [145]. The catalysts are characterized by a three-dimensional structure and high activity in photocatalytic CO_2_ conversion into energy and chemicals. The effect of different valence states of Cu on the photocatalytic activity of the synthesized composite was investigated. CuZnOCell-T showed higher activity than CuO-ZnOCell-T. The use of cellulose significantly improved the catalytic properties of the developed composites.

Li et al. synthesized Pd nanoparticles supported on perfluoroalkyl-modified cellulose using the solid-state method [146]. The developed palladium nanocatalyst was successfully tested in the reduction reaction of nitrobenzene to *N*-phenylhydroxylamine under mild conditions (at room temperature and in an aqueous medium). The authors of the study explained that the high efficiency of the palladium catalyst is due to the small size of palladium nanoparticles (1.0 and 2.5 nm) and the presence of F-containing groups. The surface modified with F-containing groups is highly hydrophobic, which is favorable for organic reactions in an aqueous environment. Modified cellulose with a more hydrophobic surface can lead to the adsorption of nitrobenzene on *N*-phenylhydroxylamine, thus preventing its complete hydrogenation to aniline.

Bahsis et al. used cellulose extracted from date palm as a support for the immobilization of copper (II) and copper (I) ions [147]. The developed copper catalysts demonstrated high efficiency in the synthesis of 1,4-disubstituted-1,2,3-triazoles at room temperature in an aqueous medium. Biocatalysts are easily separated from the reaction mixture and can be reused at least five times without a significant decrease in catalytic properties.

Prekob et al. immobilized palladium nanoparticles on carbonized cellulose by using the impregnation method [148]. The results of the catalyst physicochemical study confirmed the formation of Pd nanoparticles. The nanocatalyst was studied in the hydrogenation reaction of nitrobenzene in a methanol medium. The hydrogenation reaction was carried out at different temperatures (283–323 K) and constant hydrogen pressure (20 bar). The optimum process conditions were 303 K and 20 bar pressure. The maximum conversion of the substrate reached >99%.

Dohendou et al. presented a review on the use of biopolymers—including cellulose—as supports in the design of catalysts for the Heck cross-coupling reaction [36]. It was shown that catalytic systems based on natural polysaccharides are characterized by high sorption capacity, activity, and stability, which makes it possible to use natural polymers as supports in the synthesis of Heck cross-coupling reaction catalysts.

Fan et al. developed new bimetallic catalysts for the Sonogashira reaction by attaching polyethyleneimine (PEI) to the end of the PAMAM dendrimer grafted to cellulose, followed by the sorption of Pd(II) and Cu(II) ions [149]. Tree-shaped PAMAM was chosen as a support for the introduction of palladium and copper ions. PEI and cellulose with a large number of reactive OH- groups were used to increase the copper content in the catalyst. The synthesized bimetallic catalyst showed high efficiency in the synthesis of alkynes and benzofurans via the Sonogashira reaction and could be reused for up to seven consecutive cycles without significant loss of activity. The activity of the obtained catalyst was comparable to that of already-known catalytic systems.

Dewan et al. proposed the use of cellulose extracted from pomegranate peel waste as a support for Pd(0) nanoparticles [150]. This nanocatalyst was investigated in Suzuki–Miyaura and Sonogashira cross-coupling processes under mild conditions. The presence of sites in the cellulose matrix appropriate for interaction favored the uniform distribution of palladium nanoparticles on the support surface. The catalyst could be easily separated from the reaction mixture.

In summarizing the above, the use of cellulose in the design of nanocatalysts as a support and stabilizing agent brings a number of advantages: the increased dispersion of active phase nanoparticles, stability, reusability, etc.

### 4.2. Cellulose-Based Magnetic Catalysts

The functionalization of magnetic nanoparticles using polymers provides magnetic catalysts with improved characteristics by varying the nature of the polymer and reducing agent, the ratio of components, etc. Cellulose has a clear morphology, high specific surface area, and chelating ability, which makes it attractive for the functionalization of magnetic particles. The studies presented in [151,152,153,154] are devoted to the use of cellulose in the synthesis of magnetic nanocatalysts.

Zhang et al. [151] developed magnetic CuFe_2_O_4_@Ag@ZIF-8 nanospheres deposited on cellulose nanocrystals (CNCs). The catalyst included a paramagnetic core (CuFe_2_O_4_@Ag) and a porous shell in the form of a zeolite framework (ZIF-8). CuFe_2_O_4_ NPs were prepared in the presence of dispersant and cellulose nanocrystals. Ag NPs were deposited on CuFe_2_O_4_/CNC via in situ reduction. While investigating the catalytic properties of the synthesized composites in the reduction of 4-nitrophenol, the cellulose-supported composites were found to have higher reactivity than the nanocomposite without polysaccharide and a zeolite framework (CuFe_2_O_4_@Ag). Due to the combined properties of ZIF-8 and the CuFe_2_O_4_/CNC@Ag components, the nanocatalysts exhibited high activity.

Almajidi et al. [152] synthesized nanocomposites based on cellulose, β-cyclodextrin (BCD), graphene oxide (GO), and copper and iron oxides (Cu_2_O and Fe_3_O_4_). The Fe_3_O_4_ magnetic NLCs were prepared using an in situ method. The catalytic ability of the developed nanocomposite was demonstrated in a Hantzsch reaction, with a high yield of 1,4-dihydropyridine derivatives under mild conditions. According to the authors of the work, the advantages of the new nanocatalytic system are high catalytic properties, environmental friendliness, and easy recovery.

El Allaoui et al. used a coprecipitation method to synthesize new heterogeneous catalysts based on cellulose beads (CBs) and cobalt ferrite nanoparticles (CoFe_2_O_4_ NPs) for the degradation of rhodamine B (RhB) [138]. The degradation rate of rhodamine B reached 98.51% at 25 °C. The developed CoFe_2_O_4_@CB catalyst showed high stability and the possibility for reuse. The obtained results indicate the promising application of mechanically obtained CoFe_2_O_4_@CB catalysts in wastewater treatment.

**Figure 40:**
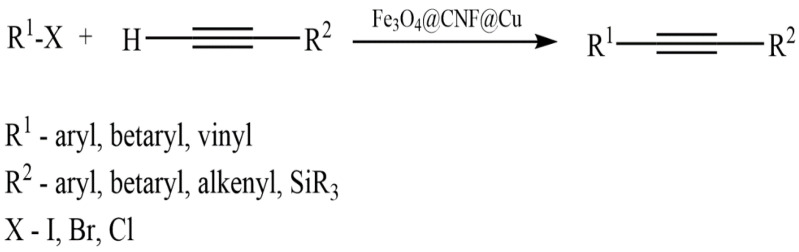
Sonogashira cross-coupling reaction.

Ghamari Kargar et al. synthesized copper/cellulose-modified magnetite for use as a catalyst in Ullmann synthesis and Sonogashira cross-coupling reactions in an aqueous medium [153]. The synthesis of the hybrid composite involved the sequential immobilization of copper nanoparticles on cellulose-modified magnetite. The advantages of this development were the ease of separation of the catalyst from the reaction mixture using an external field and the possibility of its reuse up to six times without a significant loss of catalytic activity. The figure shows the general scheme of the Sonogashira reaction, which is the cross-coupling of aryl or vinyl halides with terminal alkynes to generate conjugated enynes and arylalkynes (Figure 40).

**Figure 41:**
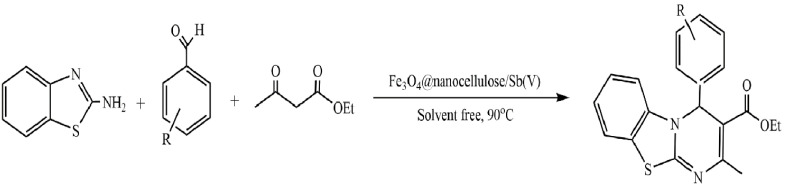
Synthesis of 4-*H*-pyrimido[2,1-b]benzothiazole derivatives in the presence of Fe_3_O_4_@nanocellulose/Sb(V) under solvent-free conditions at 90 °C.

Hosseinikhah et al. developed a magnetic nanocatalyst: Fe_3_O_4_@@nanocellulose/Sb(V) [154]. The composites were prepared using the in situ coprecipitation of Fe^3+^ and Fe^2+^ ions with ammonium hydroxide in an aqueous suspension of nanocellulose. The obtained composite was treated with a SbCl_5_ solution. The catalytic properties of the synthesized composite were successfully tested in the synthesis of 4*H*-pyrimido[2,1-b]benzothiazole derivatives via a three-component reaction between aromatic aldehyde, 2-aminobenzothiazole, and ethylacetoacetate (solvent-free) at 90 °C (Figure 41). It was found that the nanocellulose-based magnetic catalyst is easily recyclable and can be reused at least three times without significant loss of activity.

Thus, functional nanocomposites based on magnetic nanoparticles and cellulose demonstrate good properties due to a combination of magnetic and catalytic advantages and are attractive for use in catalysis.

### 4.3. HEC-Based Nanocomposites

One of the most important problems in the use of cellulose is its insolubility in various solvents because the hydroxyl groups form strong intramolecular hydrogen bonds. The strong glucoside bonds in cellulose also increase its stability in water, organic solvents, etc. In order to solve this problem, some modifications to cellulose must be made [36].

**Figure 42:**
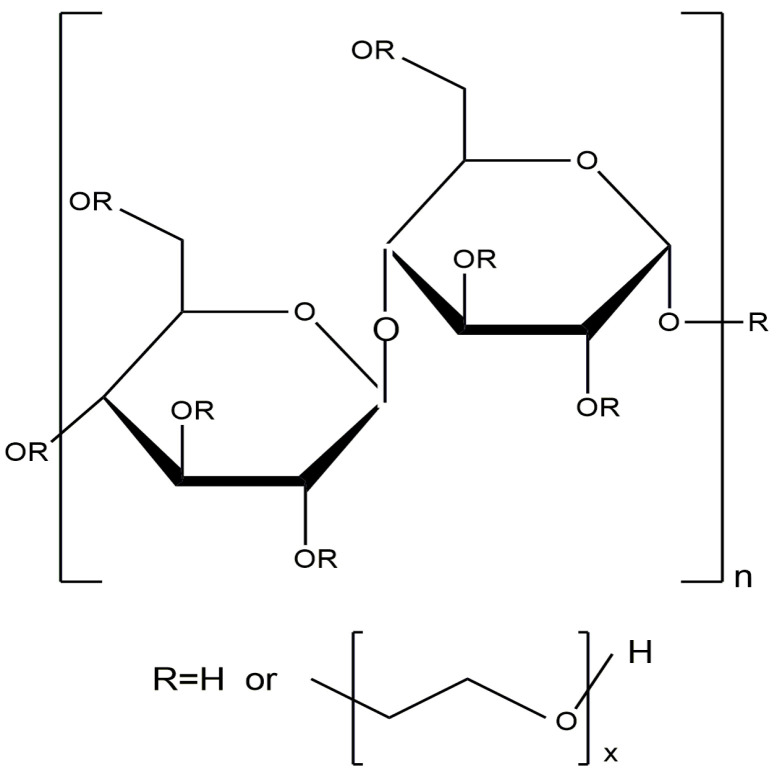
Structural formula of an HEC monomer.

Hydroxyethyl cellulose (HEC) is one of the most important commercially available cellulose derivatives and is a kind of nonionic cellulose ester with good solubility in water, unlike cellulose [155,156]. HEC is formed when cellulose is treated with an alkali and reacts with ethylene oxide [157]. In the HEC molecule, hydroxyethyl (-CH_2_-OH) groups are connected to glucopyranose monomers (Figure 42). Hydroxyethyl groups promote the formation of uniformly dispersed solutions in water; this imparts properties such as biocompatibility, hydrophilicity, and customizability [156].

Due to the above features, HEC is actively used as a binding agent, thickener, stabilizer, water retaining agent, etc. [158,159,160]. Some recent studies have reported the use of HEC in the design of heterogeneous catalytic systems [22,158,161]. By having a large number of reactive OH- groups on its chains, HEC can act both as a reducing agent for transition metal ions and as a stabilizer of metal nanoparticles.

**Figure 43:**
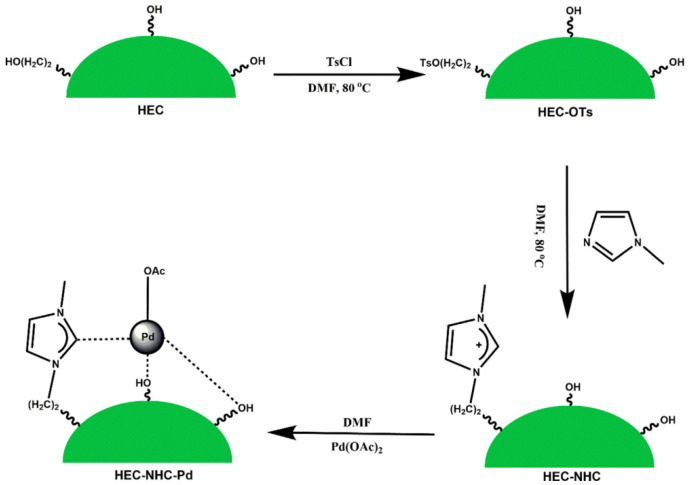
Synthetic schemes for the preparation of HEC-NHC-Pd [158].

Dong et al. developed an HEC-modified palladium catalyst for a Suzuki reaction in a water–ethanol medium [158]. The preparation method included the following stages: (1) the preparation of the intermediate of HEC-OTs by esterification in a dimethylformamide (DMF) medium; (2) the reaction of HEC-OTs with N-methylimidazole to form HEC-NHC, where NHC is N-heterocyclic carbene; and (3) the formation of a hydroxyethylcellulose-based Pd complex via an interaction of HEC-NHC with palladium acetate (Figure 43). The developed HEC-NHC-Pd catalyst showed high stability and a low degree of metal leaching (<1%).

Grządka et al. used HEC for the modification of aluminum oxide [159]. It was shown that the introduction of HEC contributed to the stability of aluminum oxide suspensions. The effect of pH, polymer concentration, and electrolyte ionic strength, as well as the nature of surfactants (CTAB and TX-100), on the adsorption and stability of the HEC/Al_2_O_3_ system was investigated. The developed systems can find wide applications in the design of various adsorbents and catalysts.

**Figure 44:**
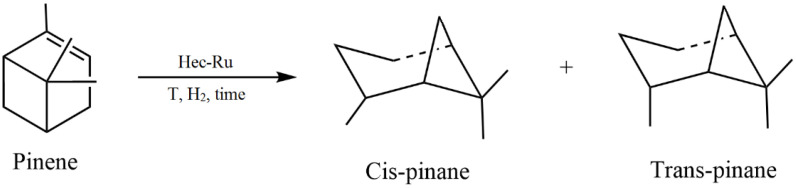
Hydrogenation of α-pinene to cis-pinane and trans-pinane.

In [161], ruthenium nanoparticles (4–6 nm) stabilized by hydroxyethylcellulose were synthesized. The use of hydroxyethylcellulose positively affected the stability and dispersion of ruthenium nanoparticles. The developed HEC-Ru catalysts showed activity (99.6%) and selectivity (98.6%) in the hydrogenation reaction of α-pinene in an aqueous medium (Figure 44). High values of α-pinene conversion (80%) and selectivity (98%) were achieved after 10 consecutive portions of the substrate. The high catalytic efficiency of the synthesized catalysts based on HEC-Ru nanoparticles was explained by the fact that HEC served as a “microreactor”, which can increase the contact between the substrate and catalyst by loading α-pinene into hydroxyethylcellulose micelles through hydrophobic interaction.

Unlu et al. used HEC to prepare catalytic membranes containing tungstosilicic acid hydrate (TSA), tungstosilicic acid supported on zirconium oxide (TSA/ZrO_2_), and sulfated zirconium oxide (SO_4_^−2^/ZrO_2_) as heterogeneous catalysts for the synthesis of the fuel bioadditive ethyl levulinate from levulinic acid and ethanol [162]. The inorganic components of catalytic membranes were prepared via a wet impregnation method and were then loaded into an HEC matrix using a solution-casting method. Further, the catalytic membrane, which was dried at room temperature, was cross-linked with phosphoric acid in an aqueous solution of isopropanol; it was then dried at 80 °C for 6 h. A catalytic membrane based on SO_4_^−2^/ZrO_2_ was found to be the most optimal catalyst for the synthesis of ethyl levulinate. The conversion of levulinic acid reached 89%.

**Figure 45:**
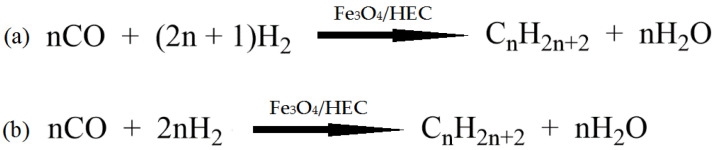
Fischer–Tropsch synthesis to paraffins (**a**) and olefins (**b**).

Zhang et al. used hydroxyethyl cellulose and carboxymethyl cellulose (CMC) in the synthesis of Fe_3_O_4_/HEC and Fe_3_O_4_/CMC catalysts [136]. Fe_3_O_4_ was prepared by using a solvothermal method and then modified with polysaccharides (HEC and CMC). The HEC- and CMC-modified catalysts were investigated in Fischer–Tropsch synthesis (Figure 45). Fe_3_O_4_/HEC and Fe_3_O_4_/CMC showed higher selectivity for olefins in comparison to unmodified Fe_3_O_4_. This was explained by the fact that the organic components of the catalysts prevented the secondary hydrogenation of primary olefins. The highest degree of carbon monoxide conversion was achieved in the presence of Fe_3_O_4_/CMC and was 50.8%. The activity of the CMC-containing catalyst was explained by the electronic effect of -COONa in the polysaccharide.

**Figure 46:**
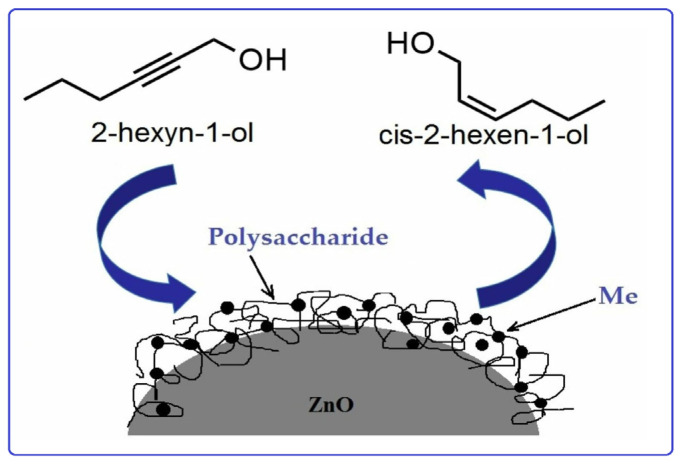
Supported on ZnO polysaccharide-stabilized palladium catalyst for the hydrogenation of acetylene compounds. Reprinted from ref. [22].

Zharmagambetova A et al. [22] proposed a simple method for the preparation of heterogeneous palladium and palladium–silver nanocatalysts containing biopolymers (HEC, chitosan, and pectin) as modifying agents and ZnO as a support material. The developed nanocatalysts were tested in the low-temperature hydrogenation of 2-hexyn-1-ol (Figure 46). The HEC-based catalyst showed the best catalytic properties (activity, selectivity, and stability) when compared with those of similar catalysts modified with chitosan and pectin.

Thus, the promising application of hydroxyethyl cellulose in the design of nano-sized catalysts for various catalytic processes is shown. The strong chelating ability of HEC toward transition metals opens the possibility of varying the metal content in catalysts and developing metal-active centers on the surface of polysaccharide-modified supports by forming polymer–metal complexes. The use of such catalysts makes it possible to carry out processes at low pressures and temperatures.

## 5. Starch-Based Nanocomposites

**Figure 47:**
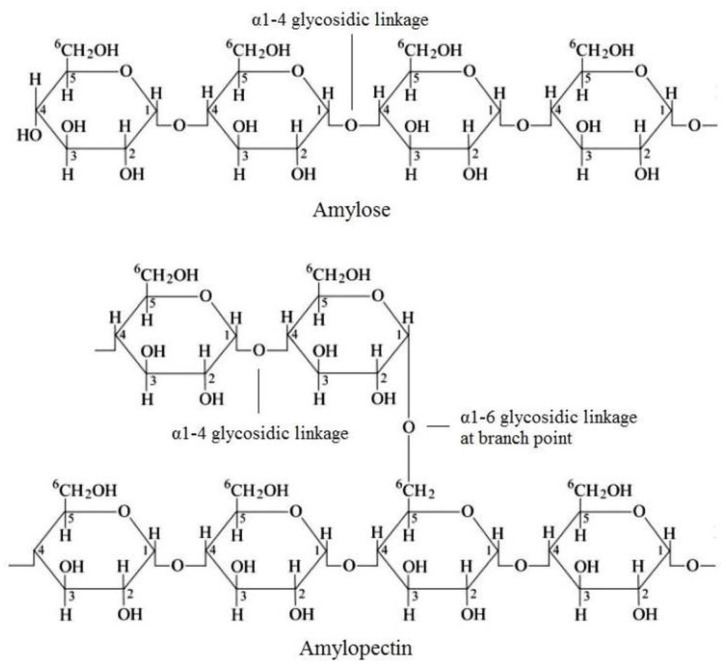
Structure of amylose and amylopectin in starch. Reprinted from ref. [165].

Starch is a carbohydrate and a natural component of most plants; it is commercially derived from grains and serves as an important raw material in various industries, such as medicine, food, and chemicals [163]. Starch’s versatility in industrial applications is largely defined by its physicochemical properties and functionality [164]. Chemically speaking, starch has the chemical formula (C_6_H_10_O_5_)_n_ and can be defined as a homopolymer of α-glucopyranose units. Starch is composed of two kinds of polysaccharide chains, amylopectin and amylose (Figure 47). Amylopectin has a branched structure with both α1-4 and α1-6 glycosidic linkages linking the monomers together. Amylose has a linear structure, with α1-4 glycosidic linkages connecting the glucose monomers [165]. In its natural state, amylose accounts for about 20–30% of starch and amylopectin accounts for 70–80% [165]. In terms of its crystallinity, size, and arrangement of polymers within the granule, starch’s structure and chemical properties are affected by the amylose/amylopectin ratio [165].

This polysaccharide presents interesting dynamic supramolecular associations facilitated by inter- and intramolecular hydrogen bonding, resulting in a helical structure, which can act as a template for metal nanoparticle growth [166]. In addition, starch can be converted into porous materials, such as aerogels or carbon, and then used for the immobilization and stabilization of metal nanoparticles [167,168,169]. All of these properties make starch a promising material for designing various types of catalysts.

### 5.1. Metal Nanoparticles Stabilized with Starch

**Figure 48:**
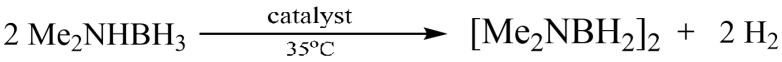
Dehydrogenation of d-DMAB in the absence of solvent.

Due to its extensive number of hydroxyl groups, starch can be used to control the growth of nanoparticles (NPs) for their use in catalysis. For example, Wongmanee et al. [166] synthesized AuNPs via the reduction of a chloroaurate anion [AuCl_4_]^−^ solution with trisodium citrate in an aqueous starch solution. The resulting starch-stabilized spherical AuNPs with a narrow particle size distribution (10 nm) were examined in terms of the homocoupling of phenylboronic acid in water using oxygen in air as an oxidant at ambient temperature (25 ± 1 °C). The catalyst exhibited good catalytic activity, with a satisfactory yield of biphenyl after being recycled for five successive runs. Duman et al. [170] described a green approach to the preparation and stabilization of metal NPs (Ru(0), Ni(0), and Cu(0)) loaded on starch in terms of their characterization and catalytic use in the dehydrogenation of dimethylamine borane (d-DMAB) in the absence of a solvent (Figure 48). Metal NPs loaded on starch were prepared (in situ) from the reduction of the acetylacetonate salts of metals and loaded on the surface of starch during the catalytic reaction (dehydrogenation of d-DMAB). All three metals were well stabilized on the surface of starch, with a particle size of about 10.0 nm, and were highly active and long-lived in hydrogen production from the dehydrogenation of DMAB at 35.0 ± 0.1 °C; they had extraordinary initial TOF and TTON values of 173 h^−1^ and 23,000 TTON for Ru(0), 130 h^−1^ and 13,000 TTON for Cu(0), and 37 h^−1^ and 4900 TTON for Ni(0) nanoparticles.

Starch-stabilized iron oxide nanoparticles for the photocatalytic Fenton degradation of methylene blue (MB) were prepared by Dasgupta et al. [171]. Two different protocols were used for their synthesis. Starch–iron (St-Fe) nanoparticles were prepared via the precipitation of ferric chloride with sodium hydroxide in the presence of starch and trisodium citrate. The second way to prepare iron–starch (Fe-St) nanoparticles was by adding cooked starch to freshly precipitated iron oxide. In both cases, the hydrophilic starch effectively prevented the precipitation of the nanoparticles under conditions closer to neutrality and allowed for the formation of a colloidal catalyst system with high activity and reusability. A mineralization level of up to 65% was achieved for 4 mg/L MB under UV irradiation at pH 4 when using 32 µmol Fe/L and 90 µmol/L H_2_O_2_ with the 100 mg Fe-St catalyst. Catalyst reusability was most notable for the 300 mg St-Fe system, which remained active in the degradation of up to seven aliquots of 4 mg/L MB in successive additions when 99 µmol Fe/L and 360 µmol/L H_2_O_2_ were used. The catalysts were also shown to be stable under acidic conditions, as no Fe^3+^ leaching was observed over a pH range of 2–5.

In [172], the hydrolysis of Co(III), Mn(III), Fe(III), and Cu(II) salts in the presence of dextrated starch resulted in polymer-stabilized colloidal hydroxides as catalysts for water oxidation:

$$H_{2}O\overset{hv}{\to }O_{2}↑+4H^{+}+4e^{-}$$

Molecular mechanics and dynamic light scattering studies indicated a core–shell-type structure for the catalysts, where the hydroxide core is stabilized by the molecular starch network (ca. 5–7 nm). The colloidal catalysts were highly efficient in oxidizing water using one electron oxidant [Ru(bpy)_3_]^3+^ at pH 7 to 10. The influence of pH, catalyst concentration, and the nature of the buffer on oxygen yield was studied. The maximal yields were 72, 53, and 78% over Fe-, Mn-, and Co-containing catalysts, respectively, and the turnover numbers were 7.8, 54, and 360, respectively. The Cu-containing catalyst showed poor effectiveness in water oxidation (the maximal yield was 28% O_2_).

**Figure 49:**
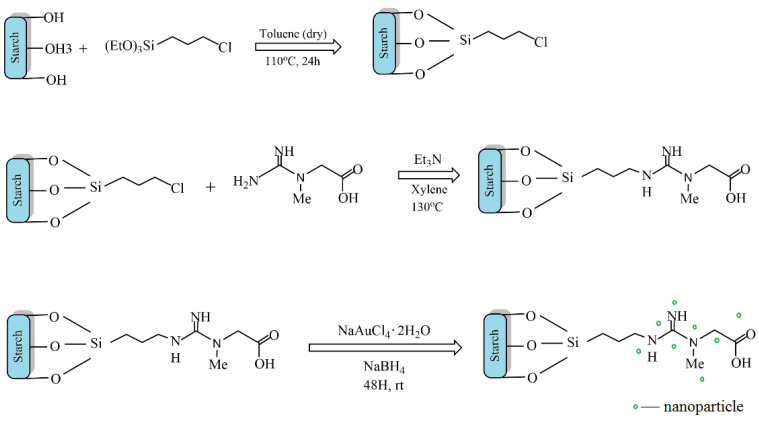
Synthesis of starch-crt@Au [173].

Starch can easily be functionalized to improve its properties, including its ability to chelate and stabilize metal nanoparticles. Gholinejad et al. [173] prepared and characterized an efficient novel catalyst comprising 4 nm Au nanoparticles supported on creatine-modified starch. For the preparation of the catalyst, commercially available corn starch was reacted with (3-chloropropyl)triethoxysilane in dry toluene, providing the resulting starch, which contained the chloro atom at the end of the linker (starch@Cl). Then, creatine was reacted with starch@Cl in xylene using Et_3_N as a base. Finally, the creatine-functionalized starch (starch@crt) was treated with NaAuCl_4_·2H_2_O and reduced with NaBH_4_, yielding creatine-modified starch supporting AuNPs (Figure 49). The obtained new gold composite demonstrated high catalytic activity in the reduction of nitroarenes into the corresponding aromatic amines with NaBH_4_ at room temperature in aqueous media (yield 89–100%). In addition, the performance of the catalyst was fully retained during its employment in several catalytic batches, and it is possible to recover it up to 12 times without a decrease in its catalytic activity. It was found that the presence of creatine in the structure of the catalyst plays a more important role in the amount of Au loading, the efficiency of the catalyst, the recycling times, and the leaching of Au compared to starch-supported Au without creatine.

**Figure 50:**
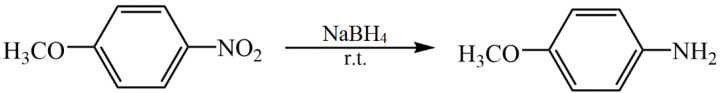
Catalytic hydrogenation of *p*-nitroanisole to *p*-anisidine with NaBH_4_ at room temperature.

In [174], a pH-responsive [2-(dimethylamine)ethyl methacrylate]-grafted maize starch composite was synthesized via free-radical polymerization in the presence of the crosslinker *N*,*N*′-methylenebisacrylamide combined with the gelatinization–ethanol precipitation method. This starch-based composite (in an optimized component ratio) showed a nanometer size and a regular spherical shape, as well as outstanding performance in pH responsiveness. Superiority was proven by the formation of a Pickering emulsion that underwent pH-induced reversible emulsification/demulsification for at least eight cycles without obvious performance decay or even morphology changes. By using this starch-based nanoparticle as a support for AuNPs, the formulated *D*-g-SNP/AuNP-stabilized Pickering emulsion system showed high efficiency (yield > 95%) in the catalytic hydrogenation of *p*-nitroanisole to *p*-anisidine with NaBH_4_ at room temperature (Figure 50). Meanwhile, the products could be isolated from the reaction system without residuals in the remnant phase. Its high effectiveness was highlighted by at least eight reaction cycles and negligible catalyst loss.

### 5.2. Starch-Based Magnetic Catalysts

Starch-stabilized nanoparticles (NPs) can act as efficient catalysts due to their small size and increased surface area for contact with reactants. At the same time, highly effective colloidal catalysts require special techniques, such as fine filtration or ultracentrifugation, to be separated from reaction mixtures after use. The promising way to avoid this disadvantage is the immobilization of starch-based nanocatalysts on superparamagnetic iron oxide particles, allowing for their easy separation using a permanent magnet.

**Figure 51:**
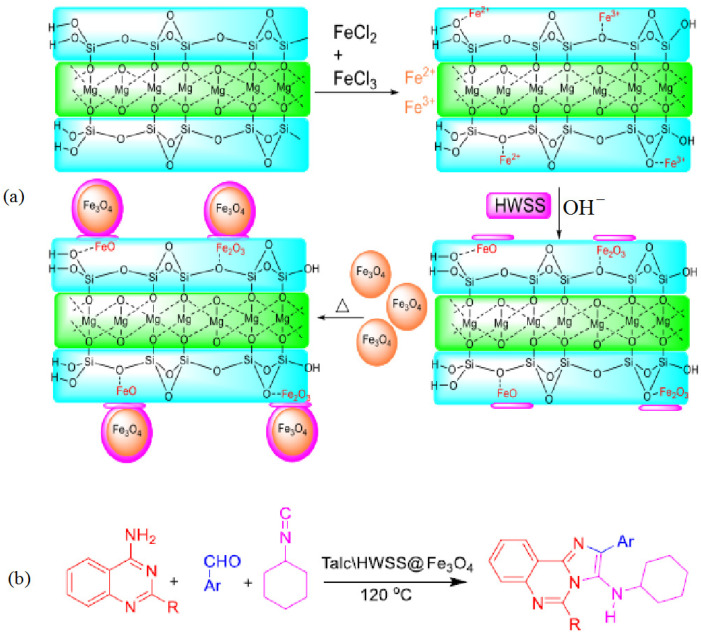
Schematic of the procedure for the preparation of the nanocomposite Talc\HWSS@Fe_3_O_4_ (**a**) and its catalytic application in a three-component reaction (**b**). Adapted from ref. [175].

Hosseinzadeh et al. [175] developed a two-step protocol to produce nano-size conglomerates of talc and magnetite nanoparticles using a hot-water-soluble starch (HWSS). Fe_3_O_4_ NPs were prepared via the coprecipitation of Fe(III) and Fe(II) oxides in an alkaline suspension of talc (Figure 51a). The addition of an aqueous solution of starch to this suspension, followed by refluxing the combined fluid, yielded the nanocomposite Talc\HWSS@Fe_3_O_4_, in which the inorganic components were adhered by thin films of starch. The nanocomposite displayed efficient catalytic activity in the synthesis of new imidazo[1,2-c]quinazoline derivatives via the Grobke–Blackburn–Bienaymé three-component reaction of 4-aminoquinazoline, arylaldehydes, and isocyanide (Figure 51b). The efficiency of the method was exemplified by synthesizing seven new products at fairly high yields (68–83%) within short reaction times (24–30 min) using a catalytic amount of the catalyst under solvent-free conditions at 120 °C. The improved catalytic activity of Talc\HWSS@Fe_3_O_4_ in comparison to that of pristine talc can be attributed to the delamination of talc by HWSS@Fe_3_O_4_ NPs and, hence, better dispersion of talc sheets in the nanocomposite. It has superparamagnetic behavior and was proven to be easily reusable after simple magnetic separation without a significant loss of its activity.

**Figure 52:**
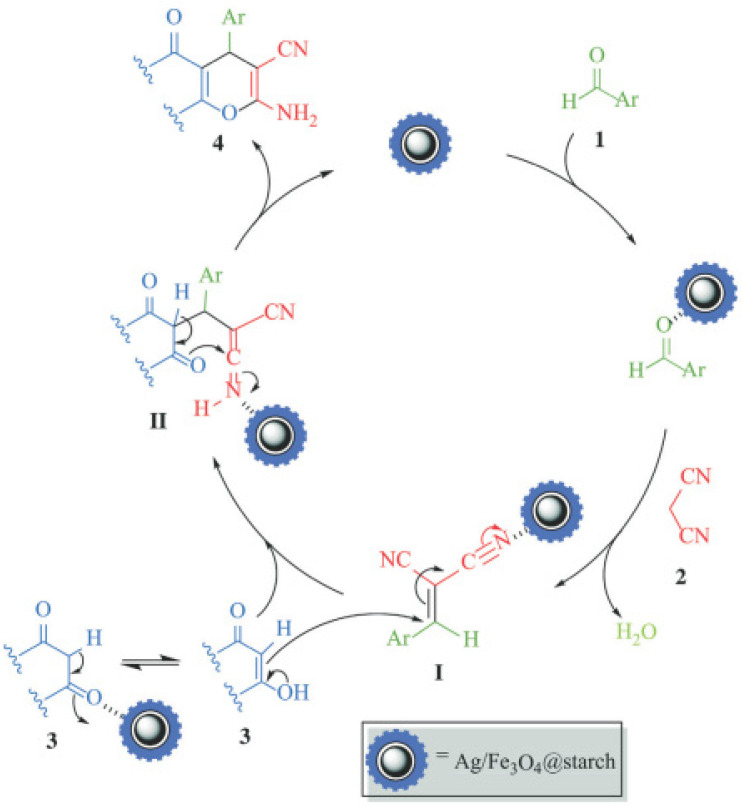
Proposed mechanism of 4*H*-pyran and tetrahydro-4*H*-chromene synthesis in the presence of a Ag/Fe_3_O_4_@starch magnetic nanocatalyst. Reprinted from ref. [176].

Mohammadzadeh et al. [176] reported that silver supported on a Fe_3_O_4_/starch nanocomposite (Ag/Fe_3_O_4_@starch) was shown to be an efficient nanocatalyst for the one-pot synthesis of 4*H*-pyrans and tetrahydro-4*H*-chromenes with 84–95% substance yields via a three-component condensation of aryl aldehydes, malononitrile, and 1,3-diketoesters or cyclic 1,3-magnetic diketones in the presence of ethanol at 50 °C (Figure 52). The nanocatalyst was easily recovered and reused with high catalytic activity, even after up to five runs.

**Figure 53:**
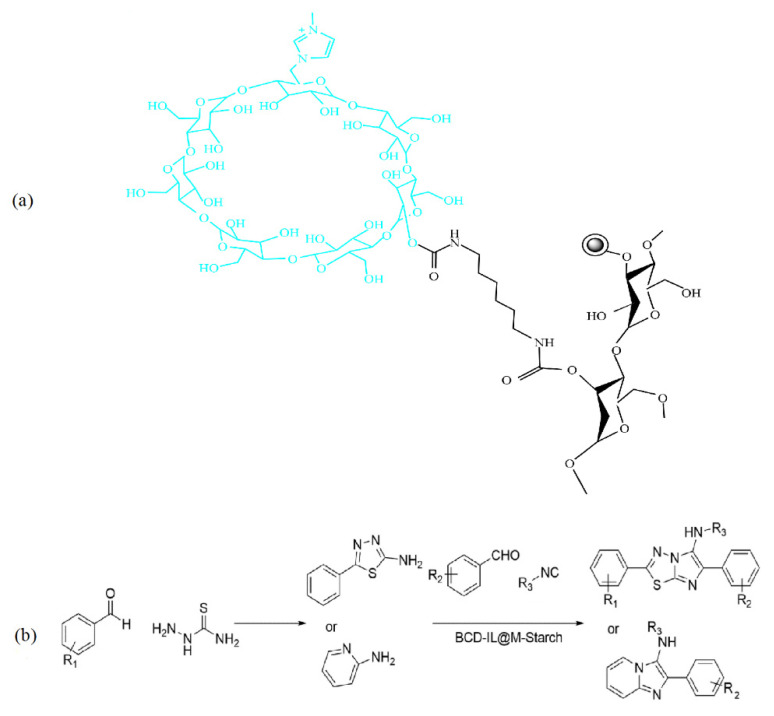
Suggested structure of the βCD-IL@M-Starch-catalyzed (**a**); synthesis of imidazo[2,1-b][1,3,4]thiadiazol-5-amine derivatives (**b**) [177].

In [177], a novel catalyst was introduced based on a β-cyclodextrine ionic liquid (βCD-IL). The βCD-IL was supported on magnetic starch for simple isolation from the reaction mixture and also for the recovery of the catalyst. For this purpose, superparamagnetic iron oxide nanoparticles (SPIONs) were first prepared via the coprecipitation of Fe^2+^ and Fe^3+^ ions. The SPIONs were then treated with starch to yield magnetic starch (M-Starch). βCD-IL was separately synthesized in two steps. The first step was a tosylation reaction using β-cyclodextrine, and the second one contained the reaction of the tosylated β-cyclodextrine with methyl imidazole. The final βCD-IL@M-Starch catalyst was prepared via the polymerization reaction of βCD-IL with hexamethylene diisocyanate on M-Starch (Figure 53a). The catalyst was used for the synthesis of imidazo[2,1-b][1,3,4]thiadiazol-5-amine and imidazo[1,2-a]pyridin-3-amine derivatives (Figure 53b). The βCD-IL@M-Starch catalyst showed very good activity in the synthesis of diphenylimidazo[2,1-b][1,3,4]thiadiazol-5-amine derivatives from the corresponding benzaldehyde, semicarbazide, benzaldehydes, and isocyanides. The products were obtained under mild reaction conditions (25–50 °C) with good isolated yields (17–84%). The catalyst was shown to be magnetically reusable and gave very good results in 10 sequential reactions.

### 5.3. Starch as Carbon Precursor in the Synthesis of a Carbon-Based Catalyst

The development of a novel approach for the generation of a mesoporous carbonaceous material, termed Starbon^®^, which possesses a large surface area ranging from 150 up to 500 m^2^/g and is obtained via the pyrolysis of mesoporous expanded starch through gelatinization, retrogradation, and dehydration processes, has become another example in favor of using this polysaccharide in the design of heterogeneous catalysts [178].

**Figure 54:**
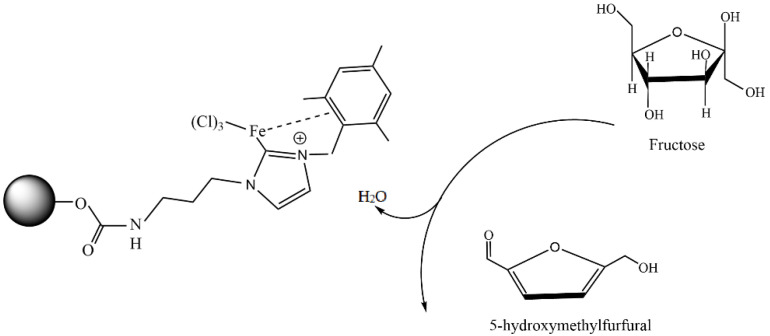
Iron–nitrogen heterocyclic carbene (Fe-NHC) on HACS and S350, catalyzing the dehydration of fructose to 5-(hydroxymethyl) furfural [179].

For example, Pt, Pd, Rh, and Ru nanoparticles supported on Starbon^®^ were shown to be efficient catalysts for the hydrogenation of succinic acid [179]. Matharu et al. [179] reported that iron–nitrogen heterocyclic carbenes (Fe-NHCs) immobilized on both mesoporous expanded starch (HACS) and Starbon^®^ 350 (S350) can be used as efficient catalysts in the dehydration of fructose to 5-(hydroxymethyl) furfural (HMF) (Figure 54). Both catalyst types showed good performance when the reaction was explored at 100 °C and various times (10 min, 20 min, 0.5 h, 1 h, 3 h, and 6 h); Fe-NHC S350 had the highest HMF yield at 81.7% (t = 0.5 h), with TOF = 169 h^−1^, a fructose conversion of 95%, and a HMF selectivity of 85.7%; Fe-NHC-expanded HACS had the highest yield at 86% (t = 0.5 h), with TOF = 206 h^−1^, a fructose conversion of 87%, and a HMF selectivity of 99%.

**Figure 55:**
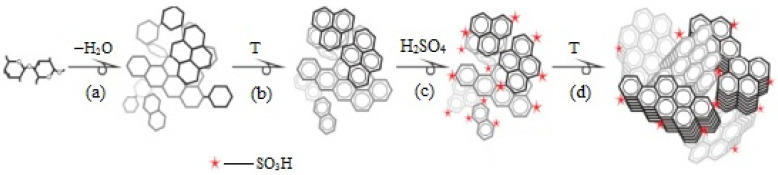
Mechanism of sulfonated carbon-based solid acid catalyst synthesis: (**a**) the removal of water from the starch; (**b**) the dissociation of the (-C-O-C-) bond, forming polycyclic aromatic carbon sheets; (**c**) the sulfonation of the carbon sheets with sulfuric acid; (**d**) carbonization with the formation of larger layered polycyclic aromatic sheets. Adapted from ref. [180].

Starch can also be used in the design of sulfonated carbon-based solid acid (CBAS) catalysts, the synthesis of which includes several steps. In the first stage, the carbohydrate is carbonized, which leads to the removal of water from the molecules and the dissociation of the C-O-C bond, forming polycyclic aromatic carbon sheets. The carbon sheets formed are then subjected to sulfonation with sulfuric acid; the acid sulfonates the aromatic rings, introducing –SO_3_H, –COOH, and –OH groups at the edge of the carbon rings. Finally, the carbonization temperature is raised again and causes the formation of the larger polycyclic aromatic sheets, along with layered polycyclic aromatic rings that reduce the number of vacancy sites for the acidic groups to attach to [165]. The mechanism of sulfonated carbon-based solid acid catalyst synthesis is shown in Figure 55. Clohessy and Kwapinski [180] compared the different CBAS catalysts for biodiesel production in terms of cost per kg, preparation conditions, operating conditions, reaction times, yield, and reusability. Among the carbohydrate-derived solid acid catalysts, starch-derived catalysts were found to be the most promising for commercial use.

Wang et al. [181] reported that porous, carbon-based solid acid from starch can also be used for the degradation of chitosan to *D*-glucosamine. A high *D*-glucosamine yield of 90.5% was obtained, with 0.2 g of the catalyst, HCl:n_chitosan_ = 1.1:1.0, and a reaction time of 8 h at 80 °C.

Thus, it was shown that the physicochemical properties and functionality of starch allowed for the development of a number of methods to design organic–inorganic composites for catalytic applications in chemical processes such as nitro-compound reduction, multicomponent reactions, transesterification, hydrogenation, dehydrogenation, water oxidation, etc. For example, the extensive number of hydroxyl groups make it possible to use starch as a stabilizing agent in the preparation of colloidal (metals, metal oxides, and hydroxides) catalysts. Starch can be converted into mesoporous expanded starch with a high specific surface area through gelatinization, retrogradation, and dehydration processes. The pyrolysis of such bulking starch allows for mesoporous carbon (Starbon) to be obtained as a support for efficient hydrogenation and dehydration catalysts. It should be noted that expanded starch can also be used in the design of supported catalysts with nearly the same catalytic properties as Starbon-based systems. Sulfonated carbon-based solid acid (CBAS) catalysts derived from starch have a high potential for commercial application in biodiesel production due to cost per kg, preparation conditions, operating conditions, reaction times, yield, and reusability. Starch can be easily modified to improve its affinity for metal ions, to graft ionic liquid as the active phase of the catalyst, and to yield catalysts with additional properties, such as pH-induced reversible emulsification/demulsification and the possibility of easy separation using a permanent magnet. We believe that the modification of starch in order to change its chelating and physicochemical properties will allow for the use of starch-based composites in catalytic applications in the future.

## 6. Conclusions

The data presented in this review indicate a growing interest in the use of natural polysaccharides in the design of composite materials for catalytic applications. This can be attributed to the fact that polysaccharides are renewable, affordable, and non-toxic polymers with suitable properties to produce efficient and reusable heterogeneous catalysts. The binding affinity of polysaccharides for transition metal ions (Pd, Au, Cu, Ag, Co, Ni, etc.) allows for the preparation of polymer–metal complexes or stabilized nanoparticles with effective catalytic properties in various reactions, such as the reduction of nitro compounds, the hydrogenation of unsaturated C-C bonds, the oxidation of C-H bonds, and the decomposition of dyes, as well as multicomponent reactions and various coupling reactions. In addition, the unique properties of polysaccharides allow for the design of composite catalysts using a wide variety of approaches.

For example, chitosan dissolved in an acidic medium to form water-soluble cationic polyelectrolyte becomes insoluble at pH > 6.3 and can be precipitated in the form of microspheres, which are used as supports for metal catalysts. This chitosan ability is also used to functionalize inorganic materials (alumina, magnetite, etc.), which contributes to the stabilization of metal nanoparticles (NPs) on their surfaces or the immobilization of nonmetal catalysts (enzymes and sulfonic acids) used in biodiesel production. The cross-linking of polysaccharide macromolecules with glutaric aldehyde is used to improve the mechanical strength of chitosan microspheres, promote the strong fixation of the chitosan shell on the surface of an inorganic support, and reduce the probability of metal NP leaching. Chitosan can also be modified to form chelate ligands (Schiff bases, etc.) with better affinity for transition metal ions, which also affects their behavior in catalytic processes. Of particular interest are the combinations of chitosan with organometallic frameworks (MOFs), where the biopolymer can act as a binding agent between MOFs and the active particles or as a support for MOF-based catalysts. Studies on the immobilization of soluble enzymes on magnetic chitosan that can provide widespread applications of enzymatic catalysts in industrial processes are also promising.

The availability and renewability of pectin has also attracted the attention of researchers as a green support for an efficient heterogeneous catalytic system. As shown in recent studies, pectin-stabilized nanocatalysts can demonstrate improved catalytic properties in various chemical transformations. Pectin promotes a decrease in the leaching of the catalyst active phase, increases the dispersibility of metal nanoparticles, and prevents their aggregation. Pectin itself is of great interest as a biocatalyst for the environmentally friendly synthesis of pharmaceuticals and biologically active compounds (e.g., dihydropyrano[2,3-c]pyrazole derivatives).

Cellulose, as well as chitosan, starch, and pectin, is an ideal support for stabilizing transition metal nanoparticles to obtain effective catalytic systems. One of the important challenges in catalysis is to obtain highly dispersed catalysts. Such catalytic systems exhibit high reactivity and reusability without a significant loss of activity. The structure of multilayer nanoporous cellulose microspheres is the most favorable for the immobilization and dispersion of metal nanoparticles. The obtained catalysts are characterized by a 3D porous structure with a high specific surface area. The LbL self-assembly method is a promising method for the design of green, active cellulose-based catalysts. Hydroxyethylcellulose (HEC), a water-soluble cellulose derivative, has great potential as an auxiliary material in the development of highly efficient catalysts. However, HEC has not yet been sufficiently studied in the design of heterogeneous catalysts.

Starch, a cheap and commercially available polysaccharide, is of equal interest. It can be converted into mesoporous expanded starch with a high specific surface area through gelatinization, retrogradation, and dehydration processes. The pyrolysis of such bulking starch promotes the formation of Starbon mesoporous carbonaceous material, which is successfully used as a support in the design of efficient catalysts for hydrogenation and dehydration processes. It should be noted that catalysts based on expanded starch have catalytic characteristics similar to those of Starbon-based systems. Sulfonated carbon-based solid acid (CBAS) catalysts derived from starch have a high potential for commercial application in biodiesel production due to their cost per kg, preparation conditions, operating conditions, reaction times, yield, and reusability. As in the case of chitosan, starch can be easily modified to improve its affinity for metal ions to graft ionic liquid as the active phase of the catalyst and to provide catalysts with additional properties, such as pH-induced reversible emulsification/demulsification and the possibility of easy separation using a permanent magnet. In our opinion, changing the chelating and physicochemical properties of starch through its modification will allow for the expansion of the catalytic applications of starch-based composites in the future.

Thus, the chemical properties of polysaccharides allow for the design of biocomposite catalysts using different methods for a variety of chemical processes. From this point of view, chitosan and starch can be used to obtain catalysts for a wide range of applications. Moreover, we also believe that the modification of cellulose and pectin can provide polymers with special properties and thereby expand the range of biocomposites for catalytic applications.
